# Supplementary material for: Low-temperature pressure-assisted liquid-metal printing for β-Ga2O3 thin-film transistors
Source: Nat Commun. 2025 Feb 22;16:1882. doi: 10.1038/s41467-025-57200-2 (PMC11846887; doi:10.1038/s41467-025-57200-2)
Supplement: Supplementary file 1 — Supplementary Information [file 41467_2025_57200_MOESM1_ESM.pdf]

Low-temperature pressure-assisted liquid-metal printing for β-Ga_2O_3 thin-film transistors

Chi-Hsin Huang[1], Ruei-Hong Cyu[2], Yu-Lun Chueh[2] and Kenji Nomura [1,3]*

[1]Department of Electrical and Computer Engineering, University of California San Diego, La Jolla, California 92093, United States

[2]Department of Materials Science and Engineering, National Tsing Hua University, Hsinchu 30013, Taiwan

[3]Material Science and Engineering Program, University of California San Diego, La Jolla, California 92093, United States

*Corresponding author: Kenji Nomura

E-mail: kenomura@ucsd.edu

Fabrication procedure for pressure-assisted liquid metal printing

Atomically thin β-Ga_2O_3 nanosheets were fabricated by pressure-assisted liquid metal printing. The process involved printing oxide skin from liquid gallium metal onto a substrate while applying uniaxial pressure. To prepare the liquid gallium metal, elemental gallium (99.99% purity, Shot Metals) was melted in a glass vial on a hot plate at 50°C in ambient air.

Supplementary Figure 1 shows the nanosheet growth procedure by pressure-assisted liquid metal printing. The setup involves a uniaxial press and hot plate in an ambient atmosphere (**Supplementary Figure 1(a)**). After the surface of SiO_2/Si substrates was cleaned by O_2 plasma (rf power = 70W for 1 min), the substrate was pre-heated before a liquid gallium droplet was placed on it. (**Supplementary Figure1(b)**) To fabricate the nanosheets, a liquid droplet of gallium metal (ranging from approximately 1 to 5mm in size) was deposited onto a SiO_2/Si substrate using a pipette. The substrate with the liquid metal droplet was then heated to a process temperature (80−200 ^{o}C), above the melting point of the liquid gallium metal (**Supplementary Figure1(c)**). During the printing process, a second substrate (also treated with O_2 plasma and pre-heated to a process temperature (80−200 ^{o}C)) was pressed onto the center of the droplet to spread the liquid alloy homogeneously between the two substrates (**Supplementary Figure1(d)**). The two substrates were then kept at process temperature (80−200 ^{o}C) and under uniaxial vertical pressure for three minutes to promote the growth of gallium oxide nanosheets (**Supplementary Figure1(e) and (f)**). After the squeezing step with uniaxial vertical pressure, the top substrate was lifted vertically and separated without any lateral slippage. Then, homogeneous ultrathin Ga_2O_3 nanosheets were exfoliated onto both substrates due to the strong van der Waals bond between the oxide skin and the substrates.

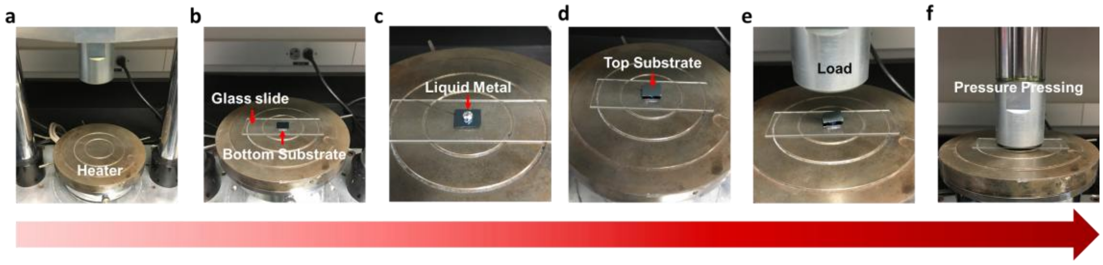

Supplementary Figure 1. Photographs of the fabrication procedure for pressure-assisted liquid metal printing under an ambient atmosphere. (a) A pressure-assisted liquid metal printing experiment setup was composed of a uniaxial press and hot plates. (b) Put the SiO_2/Si substrate on the heated glass slide. (c) Put Ga metal on top of the substrate to prepare the molten Ga. (d) Put the top substrate to sandwich the liquid metal. (e) Put down the load to apply uniaxial external pressure. (f) Growth of nanosheet with the pressure.

Reported liquid metal printing approach

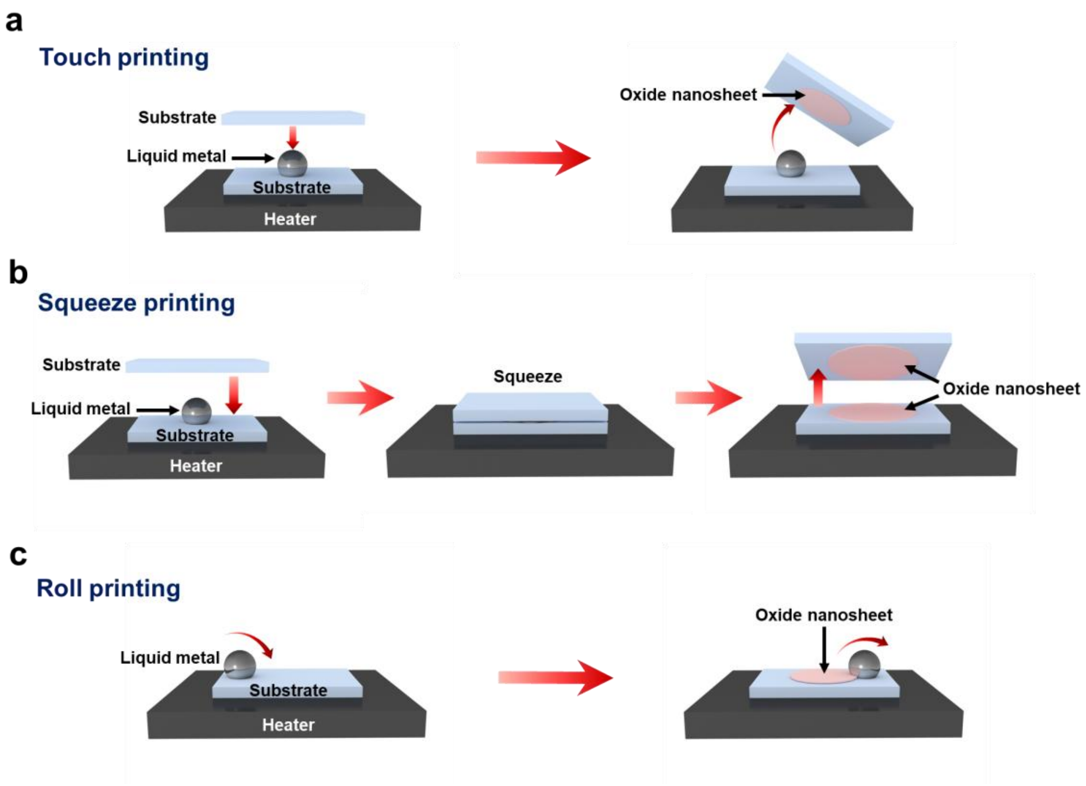

Supplementary Figure 2. Schematic of reported liquid metal printing without applying external uniaxial pressure: (a) touch printing[1, 2], (b) squeeze printing[3], and (c) roll printing[4].

Optical microscope images for large-scale ultrathin Ga_2O_3 nanosheet

Supplementary Figure 3 shows photographs and optical microscope images of large-scale (cm-sized) ultrathin Ga_2O_3 nanosheets fabricated by pressure-assisted liquid metal printing at the temperature of 150 °C under different pressures, including no pressure, a pressure of 29, 129, and 206 kPa. All of these samples exhibit large-scale Ga_2O_3 nanosheets with dimensions of around 1×1 cm^2. The entire laterally large Ga_2O_3 layer is continuous and without significant holes and cracks.

The presence of cracks or holes in Ga_2O_3 nanosheets is primarily attributed to (1) unintentional lateral forces during printing, (2) particles dropping onto the substrate from the environment, and (3) non-uniform formation of native oxide in the liquid metal. However, with careful and optimized operations, we can prepare films with good continuity and without cracks and holes, as demonstrated in **Supplementary Figure 3**.

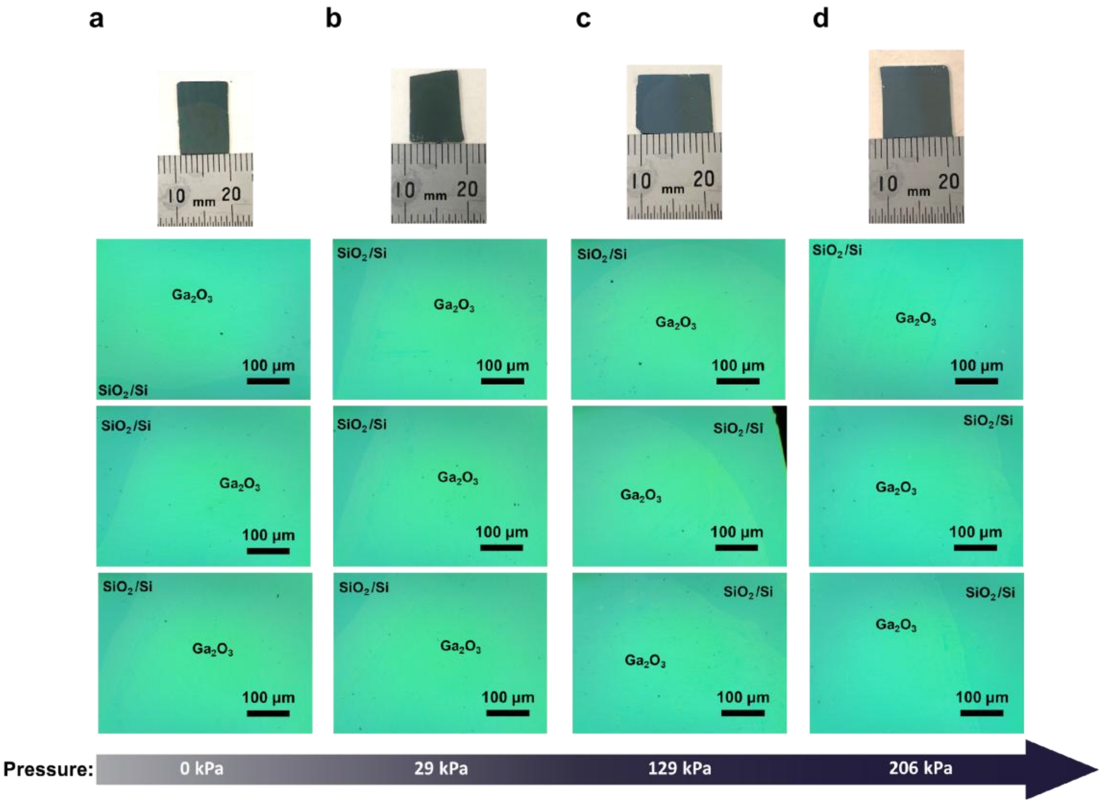

Supplementary Figure 3. Photographs and optical microscope images for large-scale (cm-sized) ultrathin Ga_2O_3 nanosheet fabricated by pressure-assisted liquid metal printing at a temperature of 150 °C under different press pressures: (a) without pressure. (b) 29 kPa, (c) 129 kPa, (d) 206 kPa.

Growth of ultrathin Ga_2O_3 nanosheet on various substrates

The developed pressure-assisted liquid-metal printing allows the direct printing of oxide materials onto various substrates. **Supplementary Figure 4** displays the optical microscope images and the photographs for the large-scale (centimeter-sized) ultrathin Ga_2O_3 nanosheets grown on various substrates, including SiO_2/Si, III-V (GaAs), transparent glass, paper, and flexible plastic substrates (PET), by pressure-assisted liquid-metal printing.

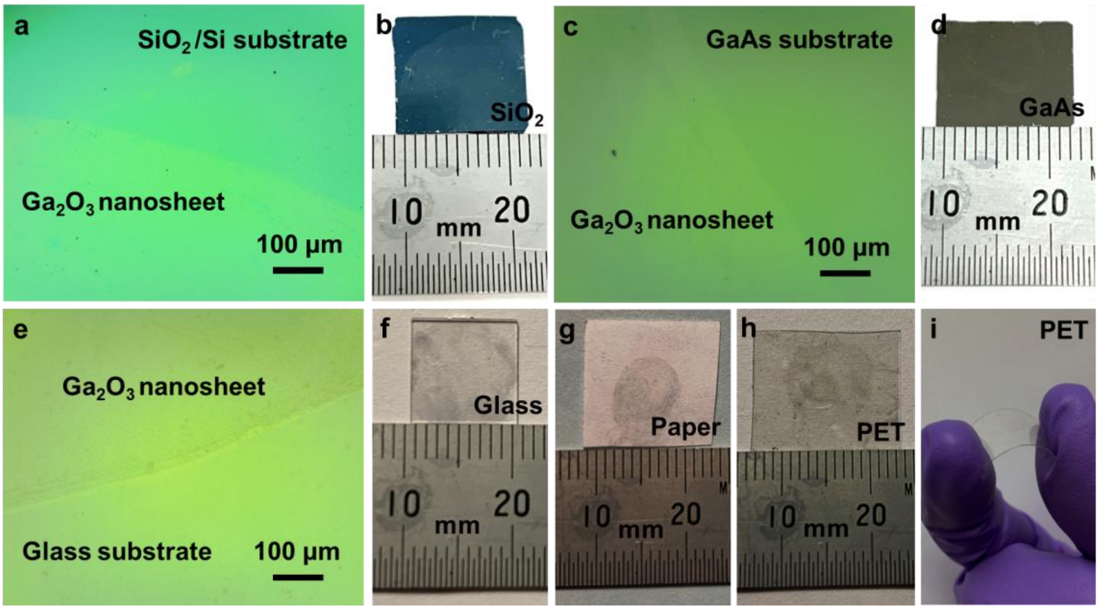

Supplementary Figure 4. Optical microscope image and photographs for large-scale (cm-sized) ultrathin Ga_2O_3 nanosheet on the various substrates fabricated by pressure-assisted liquid metal printing. (a-b) On the Si/SiO_2 substrates. (c-d) On the GaAs substrates. (e-f) On the glass substrate. Photographs image of ultrathin Ga_2O_3 nanosheet on the (g) paper, (h-i) flexible PET substrates.

Optical microscopy image and scanning electron microscopy of the Ga_2O_3 TFT

Supplementary Figure 5 shows the optical microscopy image and scanning electron microscopy (SEM) of the Ga_2O_3 TFT.

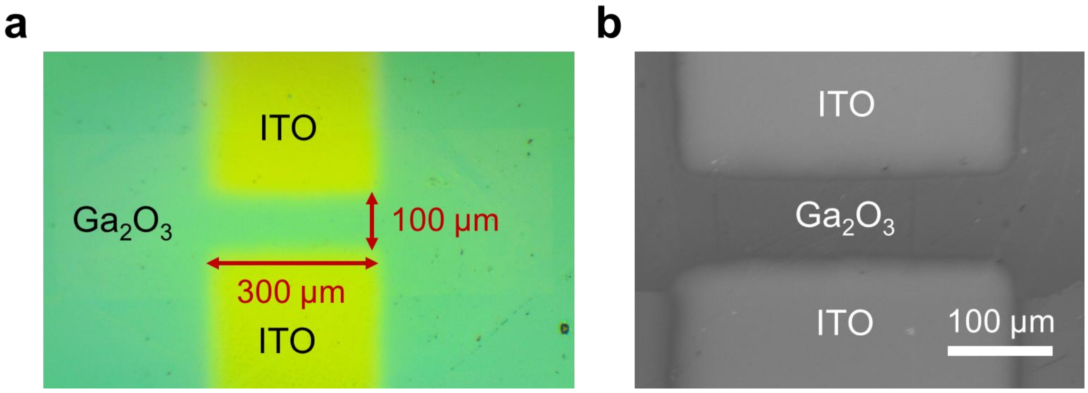

Supplementary Figure 5. (a) Optical microscopy image and (b) SEM image of the Ga_2O_3 TFT.

Summary of the TFT characteristics for the previously reported *n*-channel Ga_2O_3 TFTs

Supplementary Table1 Summary of TFT characteristics for previously reported *n*-channel Ga_2O_3 TFTs.

Material	Method	Gate Oxide	Process Temp. (ºC)	Mobility ($cm^2V^{-1}s^{-1}$)	I_{on}/I_{off}	V_{th} (V)	*s*-value (V dec.$^{-1}$)	Year	Ref.
Epitaxial Ga_2O_3 (Sn-doped)	PLD	Al_2O_3	450	$4×10^{-2}$	20	−6.7	--	2006	5
Polycrystalline β-Ga_2O_3	SP	SiO_2	700	~2	~10^4	55.8	2.72	2014	6
Amorphous Ga_2O_x	PLD	SiO_2	200	~1.5	>10^7	--	0.4	2017	7
Amorphous GaO_x	RF-MS	SiO_2	500	0.018	~10^2	−30	--	2019	8
Polycrystalline β-Ga_2O_3 (Sn-doped)	RF-MS	SiO_2	900	$0.5×10^{-3}$	~$5×10^3$	~30	--	2021	9
Amorphous GaO_x	SOL	SiO_2	500	$7.6×10^{-3}$	~10^4	13	0.16	2022	10
Amorphous GaO_x	SOL	SiO_2	500	$1×10^{-3}$	~10^4	1.7	0.34	2022	10
Polycrystalline β-Ga_2O_3	PA-LMP (low pressure)	SiO_2	80	2.6	~10^8	6	0.28	This work	
Polycrystalline β-Ga_2O_3	PA-LMP (high pressure)	SiO_2	150	11.7	~10^9	3.8	0.16	This work	

***Method**: PLD: pulsed laser deposition; SP: spray pyrosis; RF-MS: radio frequency magnetron sputtering; SOL: solution process; PA-LMP: pressure-assisted liquid metal printing.

Device-to-device statistical analysis of the presented β-Ga_2O_3 nanosheet TFT characteristic

For the demonstration of the variability of TFT performance across a substrate, **Supplementary Figure 6** shows TFT performance on a single substrate for the Ga_2O_3 TFT prepared by the P_P of 129 kPa with a T_P of 150 °C. We measured 4×4 arrays (16 devices) in the center of the sample with an area of ~10 × 10 mm^2. **Supplementary Figure 6** summarizes the histogram of TFT characteristics obtained by device-to-device statistical analysis for the working Ga_2O_3 TFTs (10 working devices). The average saturation mobility of 5.02± 4.05 $cm^2\ V^{-1}s^{-1}$, *s*-values of 0.19 ± 0.12 V·dec^{-1}, V_{th} of 3.61 ± 2.99 V, and $\log(I_{on}/I_{off})$ of 8.09 ± 1.54 were obtained.

In addition, we also provide the TFT performance on a single substrate for the Ga_2O_3 TFT prepared by the P_P of 29 kPa with a T_P of 150 °C. (**Supplementary Figure 7**) **Supplementary Figure 7** summarizes the histogram of TFT characteristics obtained by device-to-device statistical analysis for the working Ga_2O_3 TFTs (15 working devices). The average saturation mobility of 1.84± 1.34 $cm^2\ V^{-1}s^{-1}$, *s*-values of 0.38± 0.13 V·dec^{-1}, V_{th} of 7.05 ± 4.22 V, and $\log(I_{on}/I_{off})$ of 8.14 ± 0.67 were obtained. **Supplementary Figure 8** shows the batch-to-batch variation of Ga_2O_3 nanosheet TFTs. The Ga_2O_3 nanosheets were prepared using a printing a P_P of 29 kPa and a T_P of 150 °C.

The devices prepared by the higher P_P of 129 kPa show a large variation in device performance. Additionally, currently, the devices fabricated with a higher P_P of 129 kPa suffer from lower yield compared to those fabricated with P_P of 29 kPa. The optimization of the printing process, including the uniformity of pressed pressure and control of the experimental environment with controllable oxygen concentration and humidity, etc., would be important for further improvement in yield and uniformity in the future.

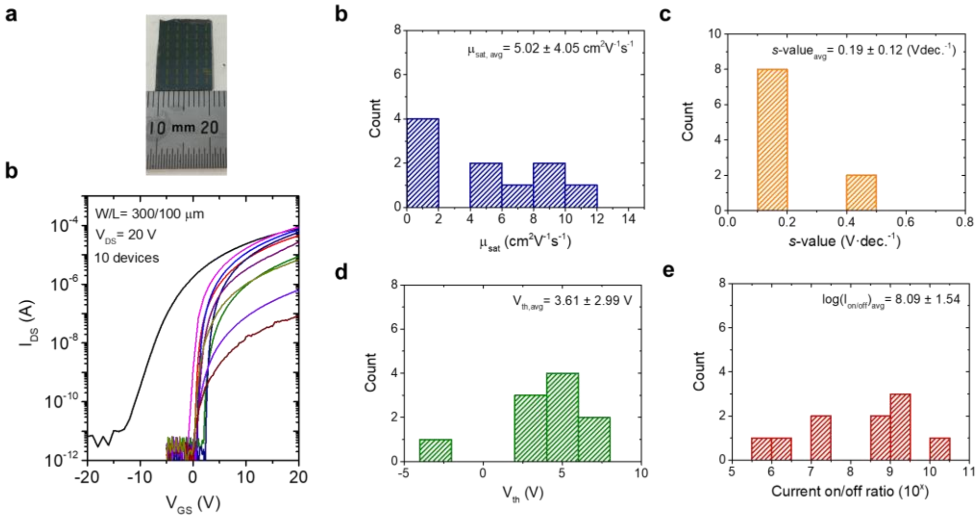

Supplementary Figure 6. (a) Photographs of the β-Ga_2O_3 TFT device. (b) Transfer characteristics for the Ga_2O_3 nanosheet TFTs for 10 devices on a single substrate. (The Ga_2O_3 nanosheet was prepared at the T_p of 150 °C and the P_p of 129 kPa) Histograms of the device performances obtained from the device-to-device statistical analysis for (c) μ_{sat}, (d) *s*-value, (e) V_{th}, and (f) on/off-current ratio, respectively.

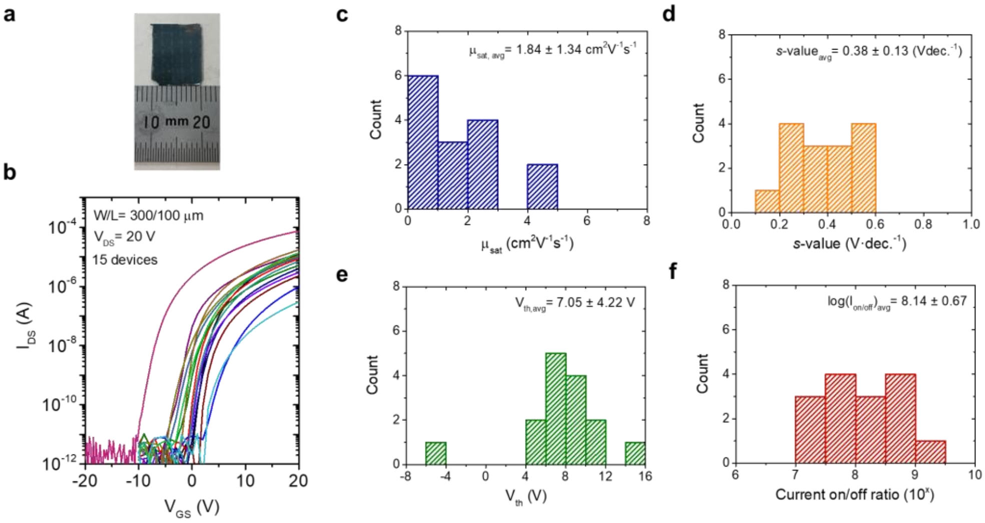

Supplementary Figure 7. (a) Photographs of β-Ga_2O_3 TFT sample. (b) Transfer characteristics for the Ga_2O_3 nanosheet TFTs for 15 devices on a single substrate. (The β-Ga_2O_3 nanosheet channel was prepared at the T_p of 150 °C and the P_p of 29 kPa.) Histograms of the device performances obtained from device-to-device statistical analysis for (c) μ_{sat}, (d) *s*-value, (e) V_{th}, and (f) on/off-current ratio of the atomically thin β-Ga_2O_3 TFT.

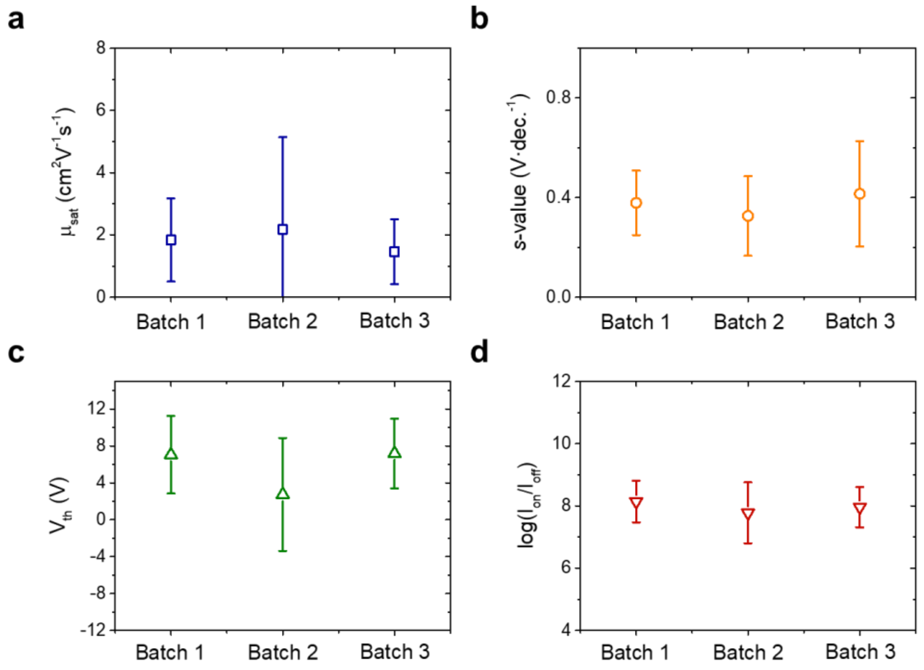

Supplementary Figure 8. Batch-to-batch variation of β-Ga_2O_3 TFTs: Ga_2O_3 nanosheets were prepared at the T_p of 150 °C and the P_p of 29 kPa. (The working devices in Batch 1 were 15, in Batch 2 were 13, and in Batch 3 were 14. The error bars are calculated using data from these respective working devices in each batch.) (a) μ_{sat}, (b) *s*-value, (c) V_{th}, and (d) on/off-current ratio, respectively.

Environmental stability of the β-Ga_2O_3 TFTs

We conducted an analysis of the environmental stability of the β-Ga_2O_3 TFTs. In **Supplementary Figure 9**, the transfer characteristics of the β-Ga_2O_3 TFTs (T_p of 150 ^oC and P_p of 129 kPa) are presented for the as-fabricated devices and those stored in ambient air atmosphere for 2 weeks and 1 month after fabrication. The results, shown for three devices each, indicate no significant degradation. We can confirm that the presented devices exhibit good environmental stability.

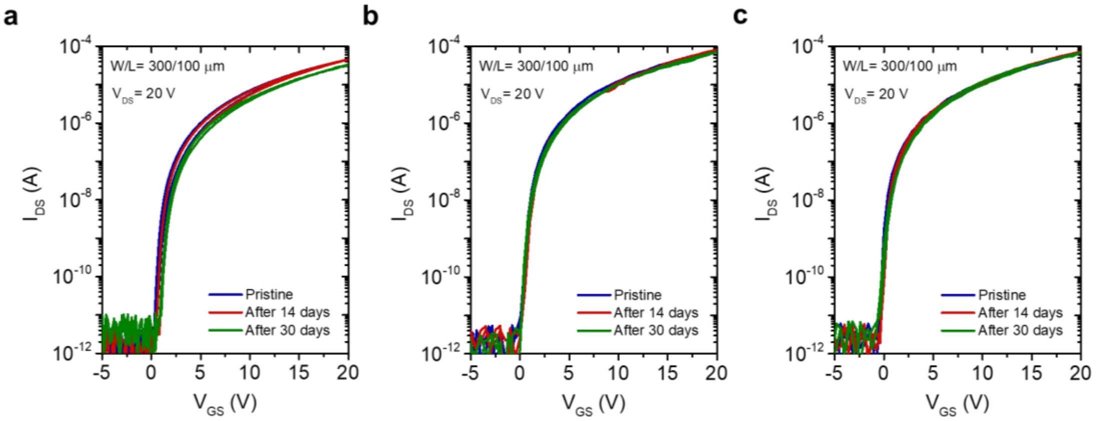

Supplementary Figure 9. Transfer characteristics for the as-fabricated β-Ga_2O_3 TFTs (T_p of 150 ^oC and P_p of 129 kPa) and those after storage in ambient air atmosphere for 2 weeks and 1 month after fabrication: (a) Device 1, (b) Device 2, (c) Device 3.

<u>Photoresponse measurements for the β-Ga_2O_3 TFTs</u>

We conducted photoresponse measurements for the β-Ga_2O_3 TFTs under optimized process conditions. (T_p of 150 °C and P_p of 129 kPa) The measurements were performed under green, blue, and UV light illuminations using the respective LEDs: green LED (λ=520 nm, Power density=0.1 mW/cm^2), blue LED (λ=460 nm, Power density=0.15 mW/cm^2), and UV LED (λ=395 nm, Power density=0.1 mW/cm^2). (**Supplementary Figure 10**) However, we observed no photoresponse under green, blue, and UV light illuminations.

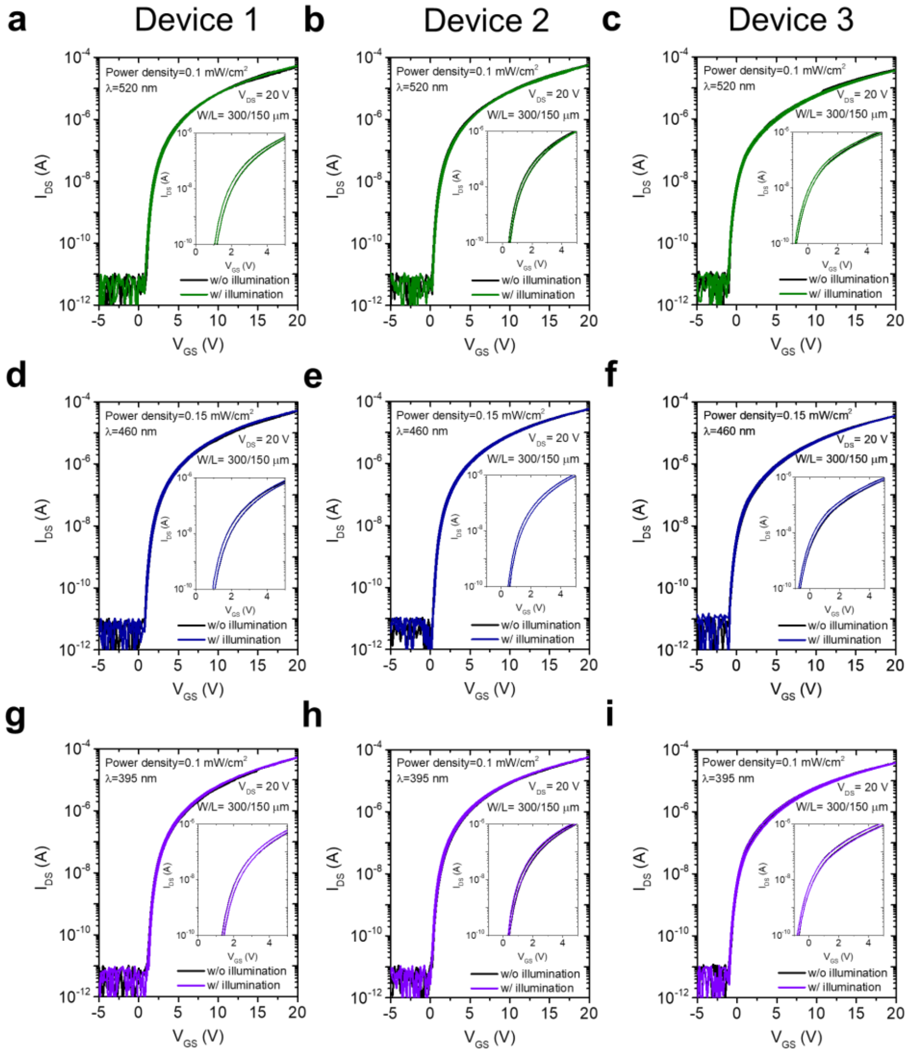

Supplementary Figure 10. Transfer characteristics for the β-Ga_2O_3 TFTs (T_p of 150 °C and P_p of 129 kPa) without and with green light illumination (λ=520 nm, Power density=0.1 mW/cm^2): (a) Device 1, (b) Device 2, and (c) Device 3. Transfer characteristics for the β-Ga_2O_3 TFTs without and with blue LED (λ=460 nm, Power density=0.15 mW/cm^2): (d) Device 1, (e) Device 2, and (f) Device 3. Transfer characteristics for the β-Ga_2O_3 TFTs without and with UV LED (λ=395 nm, Power density=0.1 mW/cm^2): (g) Device 1, (h) Device 2, and (i) Device 3.

Output conductance, transconductance, and intrinsic gain for the presented β-Ga_2O_3 nanosheet TFTs

Supplementary Figure 11 shows the output characteristics for the β-Ga_2O_3 nanosheet TFT (T_p of 150 °C and P_p of 129 kPa). The output resistance of $1/g_d$ (where g_d is output conductance, determined by $\Delta I_{DS}/\Delta V_{GS}$ in the saturation region) was > 80 MΩ (**Supplementary Figure 11 (a)**). **Supplementary Figure 11 (b)** plots the transconductance (g_m) and $1/g_d$ as a function of V_{GS}. An intrinsic gain ($A_i = g_m/g_d$) was 1,000 at V_{DS} of 30 V, which was significantly higher than that for other source-gate oxide TFTs (Schottky-barrier oxide TFTs)[11-13] and one order of magnitude higher than that for traditional ohmic contact IGZO TFTs. (**Supplementary Figure 11 (c)**)

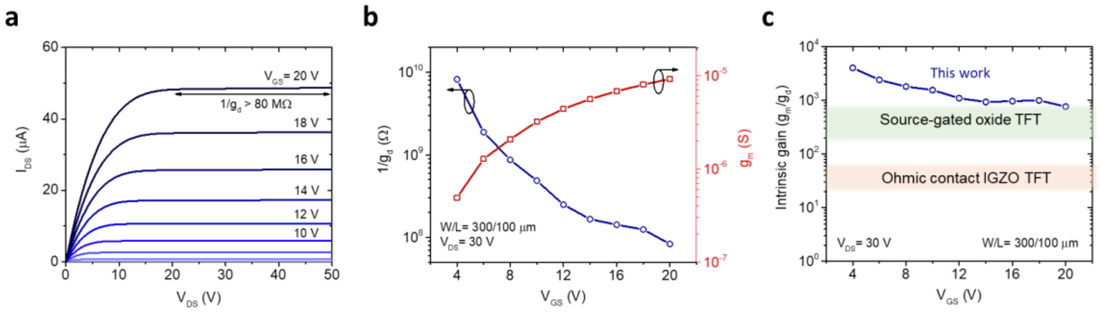

Supplementary Figure 11. (a) Output characteristics for the β-Ga_2O_3 nanosheet TFT (T_p of 150 °C and P_p of 129 kPa). (b) the extracted $1/g_d$ (blue circles) and g_m (red squares) as a function of V_{GS} at V_{DS} = 30 V. (c) Intrinsic gain, g_m/g_d, as a function of V_{GS}. The intrinsic gain for ohmic contact IGZO TFTs and source-gate oxide TFTs (Schottky-barrier oxide TFTs) are also shown for comparison.

Post-thermal annealing effect for the ultrathin β-Ga_2O_3 TFTs

Supplementary Figure 12(a) shows the variation of TFT characteristics for β-Ga_2O_3 TFTs after post-thermal annealing under an ambient air atmosphere at 100, 150, and 200 °C for 1 hour. The Ga_2O_3 nanosheet used for this test was prepared using a T_p of 150°C and a P_p of 29 kPa. The TFT characteristics remain almost unchanged during annealing up to a temperature of 200 °C. **Supplementary Figure 12(b)** shows the variation in TFT characteristics for β Ga_2O_3 TFTs after vacuum annealing (~10^{-5} mTorr) at 100, 150, and 200 °C for 1 hour. The vacuum annealing causes large negative threshold voltage shifts and off-current degradation.

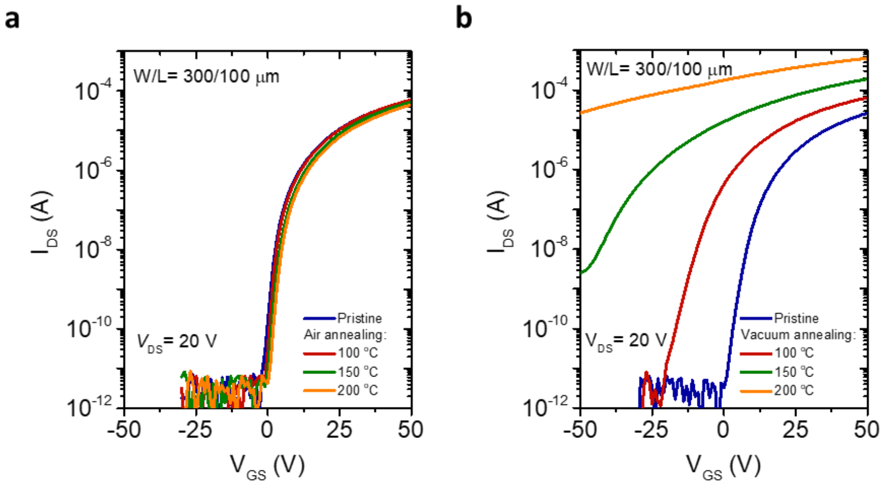

Supplementary Figure 12. (a)Variation of transfer characteristics of β-Ga_2O_3 TFTs (T_p of 150 °C and P_p of 29 kPa)by (a) ambient air annealing and (b) vacuum annealing at different temperatures for 1 hour.

TEM sample preparation procedure

First, the TEM grid was placed on top of the glass slide and pre-heated to process temperature (80−200 ^oC), which is above the melting point of the liquid Ga metal. Note that we did not perform O_2 plasma treatment for the TEM sample, as we did for the Ga_2O_3 nanosheet on the SiO_2/Si substrate.

To fabricate the nanosheets, a liquid droplet of Ga metal (with a size of <1 mm) was pipetted onto a TEM grid, and the TEM grid/glass slide with the liquid metal droplet was kept heated at 50°C to prevent the liquid Ga from solidifying. During the printing process, we used SiO_2/Si as the top substrate (pre-heated to process temperature (80−200 ^oC)), pressing it onto the center of the droplet to spread the liquid alloy homogeneously between the TEM grid and SiO_2/Si substrates. The TEM grid and SiO_2/Si substrates were kept at process temperature (80−200 ^oC) under uniaxial vertical pressure for gallium oxide nanosheet growth for 3 minutes.

After the squeezing step with uniaxial vertical pressure, the top SiO_2/Si substrate and TEM grid were carefully separated. We then used a soft wiping tool (cotton bud) to remove Ga liquid inclusions directly by gentle rubbing. It is important to note that we did not immerse the sample in ethanol, as we did for the Ga_2O_3 nanosheet on the SiO_2/Si substrate.

Following the printing process, we observed that most carbon film was broken (**Supplementary Figure 13**). During TEM analysis, we looked for nanosheets suspended and connected/supported by the bar of the TEM grid for detailed analysis (the red rectangle region in **Supplementary Figure 13**).

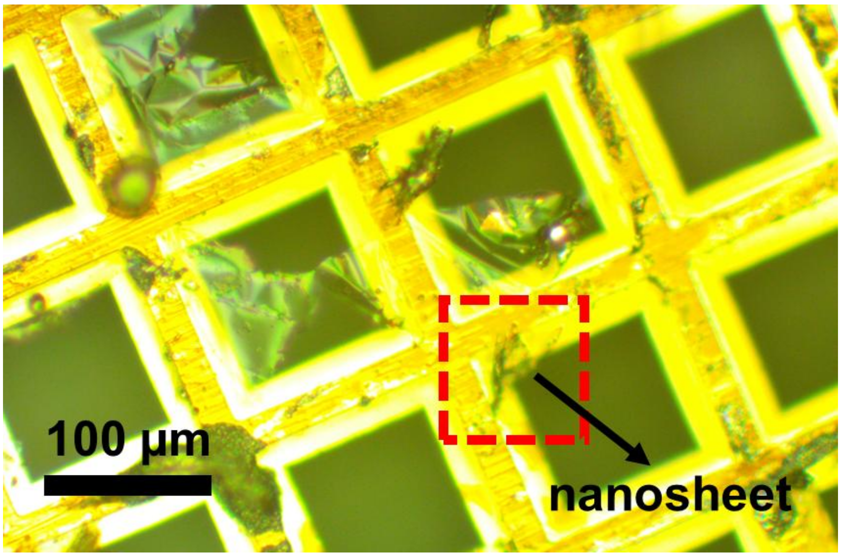

Supplementary Figure 13. Optical microscopy image of Ga_2O_3 nanosheet on the TEM grid.

Post-thermal annealing effect for amorphous GaO_x nanosheets prepared by the conventional LMP method

Supplementary Figure 14 shows the transfer characteristics of the insulating amorphous GaO_x nanosheets prepared by conventional LMP without applied external uniaxial pressure, following post-thermal annealing in different atmospheric conditions such as ambient air, vacuum (~10^{-5} mTorr), and H_2 (forming gas). No TFT actions were observed in all the devices, and the channels remained insulative even after post-thermal annealing under these different conditions.

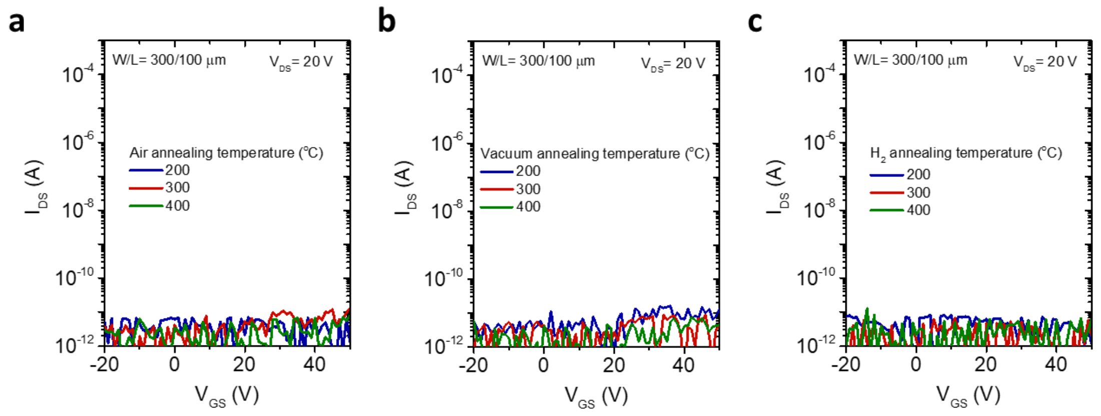

Supplementary Figure 14. Variation of transfer characteristics for the insulating amorphous GaO_x nanosheet prepared by the conventional LMP after post-thermal annealing in different atmospheric conditions, including (a) ambient air annealing at 200−400 °C, (b) the vacuum annealing at 200−400 °C, and (c) H_2 (forming gas) annealing at 200−400 °C.

TEM characterization for the Ga_2O_3 nanosheet grown by pressure-assisted liquid-metal printing

We conducted TEM analysis of the Ga_2O_3 nanosheet prepared by the pressure-assisted liquid metal printing (PA-LMP) approach, varying process parameters (**Supplementary Figure 15:** T_p of 150 °C and P_p of 129 kPa, **Supplementary Figure 16:** T_p of 150 °C and P_p of 29 kPa, **Supplementary Figure 17:** T_p of 80 °C and P_p of 29 kPa).

T_p of 150 °C and P_p of 129 kPa (**Supplementary Figure 15**):
Supplementary Figure 15(a) and (b) for the low-magnified and high-magnified TEM images of the Ga_2O_3 nanosheet. The HRTEM image also showed the crystal lattice structure with internal spacings of ~0.362 nm ~0.263 nm, which are assigned to the (201) and (−111) planes, respectively, of the monoclinic β-Ga_2O_3 crystal structure. (**Supplementary Figure 15(d)**) The corresponding selected area electron diffraction (SAED) pattern is shown in **Supplementary Figure 15(c)**, and exhibits spots indexed to the (−111), (201), (−311), and (400) crystal plans. The observation concluded that the presented nanosheet was β-Ga_2O_3 crystals.

T_p of 150 °C and P_p of 29 kPa (**Supplementary Figure 16**):
Supplementary Figure 16(a) and (b) for the low-magnified and high-magnified TEM images of the Ga_2O_3 nanosheet. The HRTEM image also showed the crystal lattice structure with internal spacings of ~0.254 nm, which are assigned to the (111) plane of the monoclinic β-Ga_2O_3 crystal structure (**Supplementary Figure 16(d)**). The corresponding SAED pattern is shown in **Supplementary Figure16 (c)**, and exhibits spots indexed to the (−111), (−113), (−313), and (400) crystal plans. The observation concluded that the presented nanosheet was β-Ga_2O_3 crystals.

T_p of 80 °C and P_p of 29 kPa (**Supplementary Figure 17**):
Supplementary Figure 17(a) and (b) for the low-magnified and high-magnified TEM images of the Ga_2O_3 nanosheet. The HRTEM image also showed the crystal lattice structure with internal spacings of ~0.282 nm, which are assigned to the (202) plane of the monoclinic β-Ga_2O_3 crystal structure (**Supplementary Figure 17(d)**). The corresponding SAED pattern is shown in **Supplementary Figure 17(c)**, and exhibits spots indexed to the (−111), (201), (−311), and (−203) crystal plans. The observation concluded that the presented nanosheet was β-Ga_2O_3 crystals.

All of these TEM analyses confirm that the Ga_2O_3 nanosheet grown under these process parameters is β-Ga_2O_3. Additionally, the TEM analysis found that high-temperature and high-pressure conditions (*i.e.* T_p of 150 °C and P_p of 129 kPa (**Supplementary Figure 15**)) produce β-Ga_2O_3 nanosheet with better crystallinity. This finding explains the observation that TFT characteristics improved when the Ga_2O_3 nanosheet was prepared under high-temperature and high-pressure conditions.

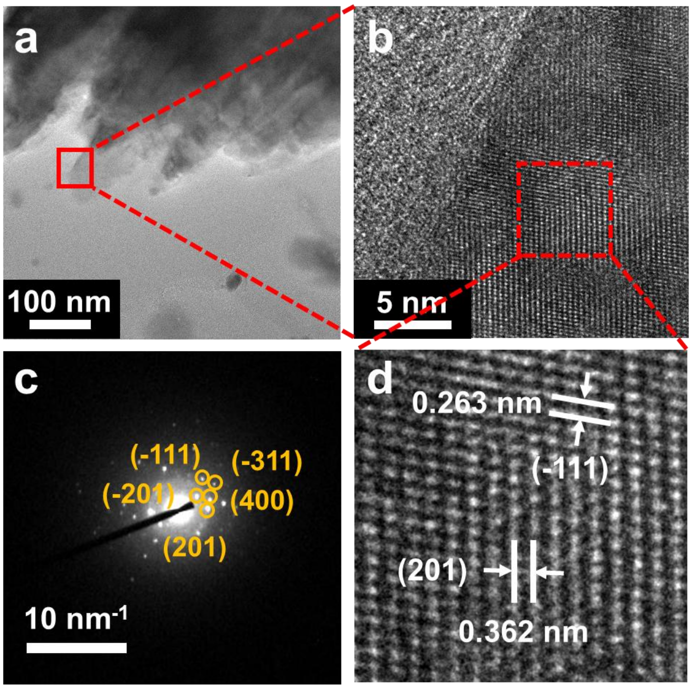

Supplementary Figure 15. TEM characterization of the Ga_2O_3 nanosheet synthesized by the PA-LMP route with the T_p of 150 °C and the P_p of 129 kPa. (a) Low-magnified HRTEM images of the Ga_2O_3 nanosheet. (b) and (d) Corresponding high-magnified HRTEM images and (c) SAED pattern.

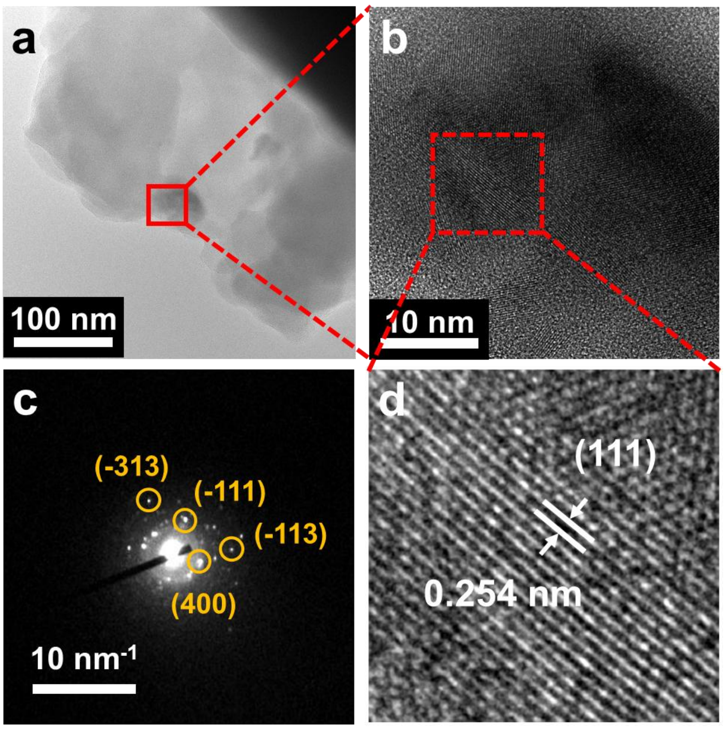

Supplementary Figure 16. TEM characterization of Ga_2O_3 nanosheet synthesized by the PA-LMP route with the T_p of 150 °C and the P_p of 29 kPa. (a) Low-magnified HRTEM images of Ga_2O_3 nanosheet. (b), (d) Corresponding high-magnified HRTEM image and (c) SAED pattern.

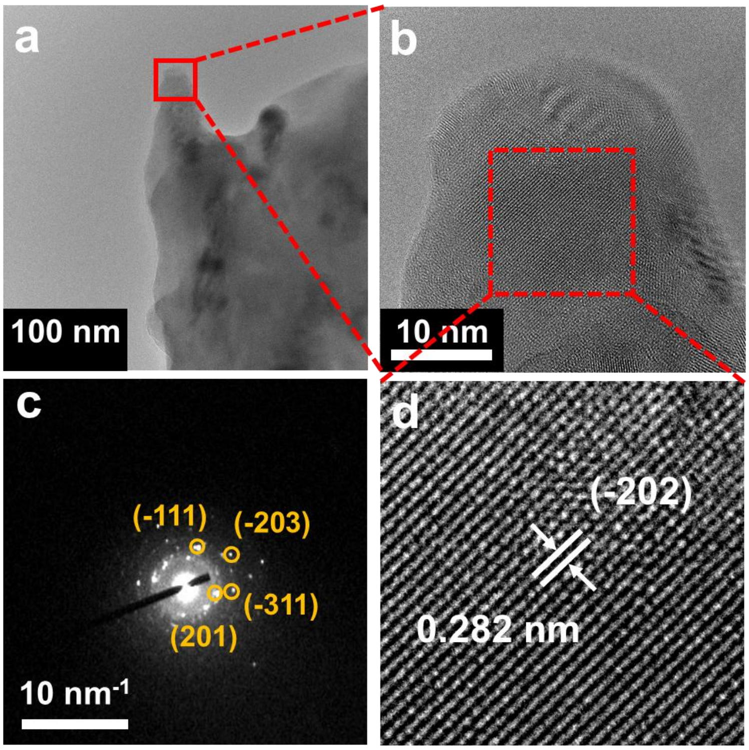

Supplementary Figure 17. TEM characterization of Ga_2O_3 nanosheet synthesized by the PA-LMP route with the T_p of 80 ^oC and the P_p of 29 kPa. (a) Low-magnified HRTEM images of Ga_2O_3 nanosheet. (b),(d) Corresponding high-magnified HRTEM image and (c) SAED pattern.

STEM and EDX characterization for the Ga_2O_3 nanosheet grown by pressure-assisted liquid-metal printing

Supplementary Figure 18 (a) shows the STEM image of the Ga_2O_3 nanosheet. (T_p of 150 ^oC and P_p of 129 kPa) The corresponding Energy dispersive X-ray spectroscopy (EDX) chemical composition mapping was performed to investigate the elemental distribution, showing that the signal intensities of Ga and O are uniformly distributed within the nanosheet (**Supplementary Figure 18 (b) and (c)**). The EDX analysis showed a Ga/O atomic ratio of 0.64, close to the atomic ratio of Ga_2O_3 (**Supplementary Figure 18 (d)**).

Besides the peaks for Ga and O, we observed peaks for C, Cu, Si, K, and Ca. The C and Cu peaks predominantly originate from the TEM grid. The Si peak may result from the materials preparation process, as we used SiO_2/Si as the top substrate for the printing process. Additionally, the Si signal might be due to cross-contamination from the tweezers. The signals of K and Ca are attributed to airborne contaminants. The entire process was conducted in a non-controlled laboratory environment, where airborne particles may contain elements like K and Ca, adhering to the sample during preparation.

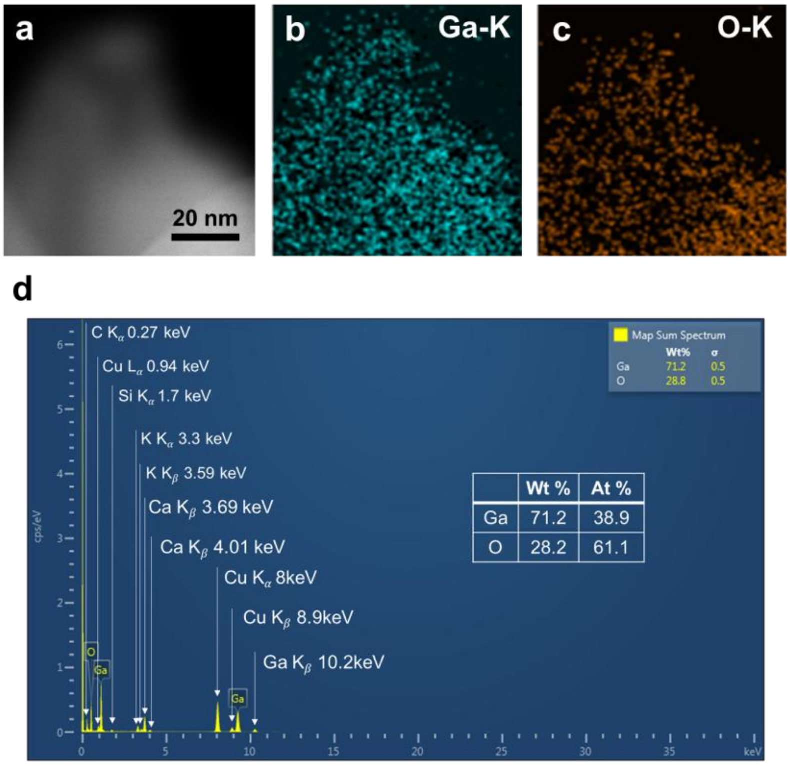

Supplementary Figure 18. (a) STEM image of the Ga_2O_3 nanosheet grown by pressure-assisted liquid-metal printing. (T_p of 150 ^oC and P_p of 129 kPa) TEM-EDX mappings for (b) Ga, and (c) O. (d) The corresponding EDX spectrum for the Ga_2O_3 nanosheet. The inset shows the atomic ratio of the Ga_2O_3 nanosheet.

XRD characterization for the Ga_2O_3 nanosheet grown by pressure-assisted liquid-metal printing

We performed grazing incidence X-ray diffraction (GIXRD) analysis for the Ga_2O_3 nanosheets prepared under different process parameters and summarized in **Supplementary Figure 19** (**green line**: LMP at the T_p of 150 °C without pressure, **blue line**: the P_p of 29 kPa and the T_p of 80 °C, **red line**: 29 kPa and 150 °C, **purple line**: 129 kPa and 150 °C, respectively). The diffraction database (PDF #01-087-1901) was used to assign the diffraction peaks. A halo peak observed around ~24° is attributed to the glass substrate. No diffraction peaks were observed for the GaO_x prepared by liquid metal printing without applied pressure (**green line**). On the other hand, Ga_2O_3 prepared by the pressure-assisted liquid metal printing method exhibited diffraction peaks assigned to the β-Ga_2O_3 crystal phase. (Note: the intensity of the diffraction peaks is weak due to the thin thickness of Ga_2O_3 being ~3nm.)

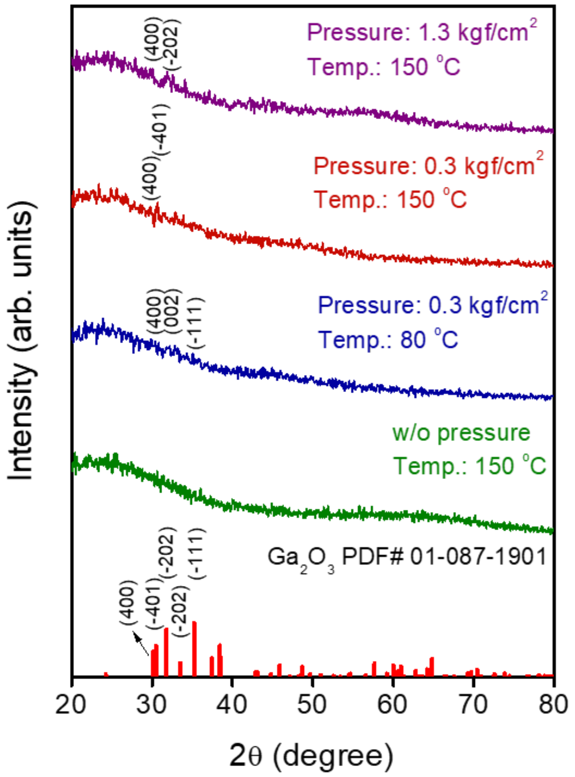

Supplementary Figure 19. GIXRD patterns for typical Ga_2O_3 nanosheet grown by PA-LMP method with different process parameters. (**blue line:** T_p of 80 °C and the P_p of 29 kPa, **red line:** T_p of 150 °C and the P_p of 29 kPa, **purple line:** T_p of 150 °C and the P_p of 129 kPa). For the comparison, Ga_2O_3 nanosheet grown by the LMP at the T_p of 150 °C without pressure was shown as **green line.** The powder diffraction file (PDF #01-087-1901) was used to identify the diffraction peaks. A halo peak around ~24° is due to the glass substrate.

Cross-sectional TEM characterization for the Ga_2O_3 nanosheet grown by pressure-assisted liquid-metal printing

We conducted cross-sectional TEM characterization for the Ga_2O_3 nanosheet prepared by the liquid metal printing approach. The Ga_2O_3 nanosheet is prepared under T_p of 150 °C and the P_p 129 kPa, exhibiting a mobility of 8~10 $cm^2V^{-1}s^{-1}$ for TFTs. In **Supplementary Figure 20(a)**, a low-magnification cross-sectional TEM image of the Ga_2O_3 nanosheet is shown. The polycrystalline nature of the Ga_2O_3 is observed. However, due to its thin thickness and weak crystallinity, it is challenging to perform reliable analysis to identify the phase of the material. Nevertheless, this result confirms the polycrystalline nature of the Ga_2O_3 over a large area.

Furthermore, we fabricated a thick Ga_2O_3 nanosheet with a thickness of ~10 nm using pressure-assisted liquid-metal printing three times, with T_p of 150 °C and the P_p 129 kPa, to perform a reliable SAED analysis. In **Supplementary Figure 21 (a)**, a low-magnification TEM image of the Ga_2O_3 nanosheet is presented. The corresponding SAED patterns from rectangle areas (1), (2), (3), and (4) in **Supplementary Figure 21**, as depicted in **Supplementary Figure 21**, were indexed to the plane of β-Ga_2O_3. These results indicate that the presented Ga_2O_3 nanosheet, prepared by pressure-assisted liquid-metal printing, is in the crystalline β-Ga_2O_3 phase over a large area.

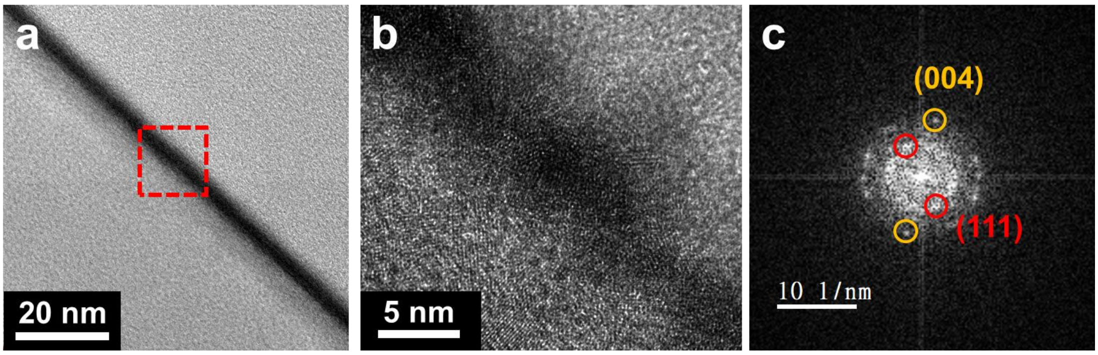

Supplementary Figure 20. (a) Cross-sectional TEM characterization of Ga_2O_3 nanosheet synthesized by the PA-LMP route with the T_p of 150 °C and the P_p of 129 kPa. (b) Corresponding cross-sectional HRTEM image and (c) corresponding diffraction pattern extracted by the fast Fourier transform.

a

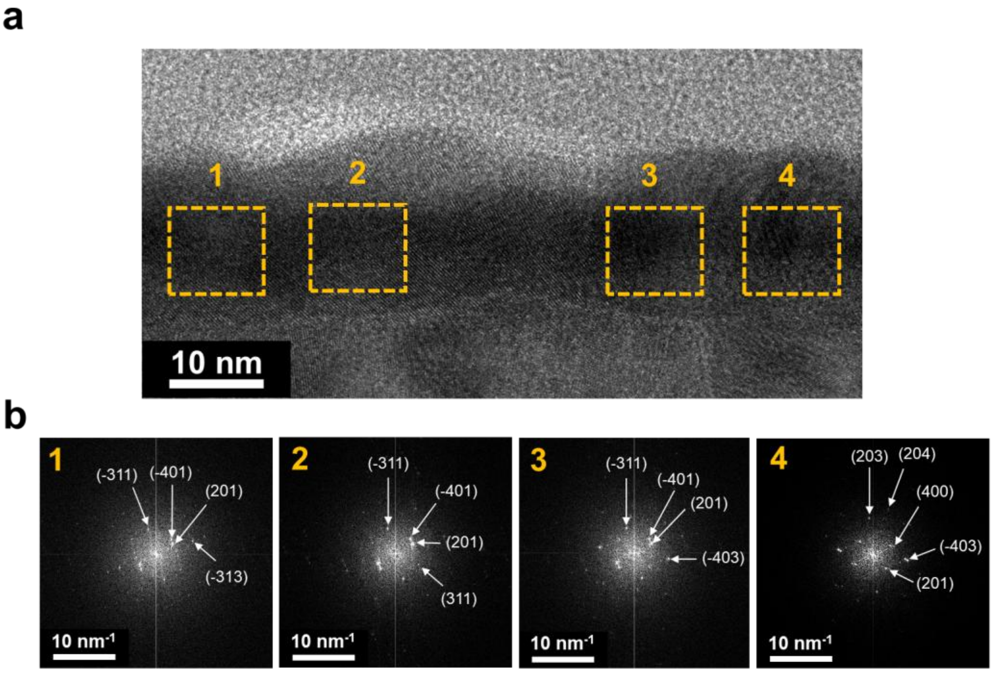

Supplementary Figure 21. (a) Cross-sectional TEM characterization of a thick Ga_2O_3 nanosheet (the thickness of approximately 10 nm) synthesized by the PA-LMP with the T_p of 150 °C and the P_p of 129 kPa. (b) Corresponding SAED pattern from rectangle areas (1), (2), (3), and (4), respectively.

To confirm the crystalline domains across the large-area thin film, we conducted cross-sectional TEM analysis at various locations, examining both crystallized Ga_2O_3 and amorphous GaOx nanosheets. **Supplementary Figure 22(a)** shows a cross-sectional low-magnification TEM image of a sample fabricated using a focused ion beam (FIB). The sample is the Ga_2O_3 nanosheet synthesized by the PA-LMP route at temperatures of 150 °C and a pressure of 1.3 kgf/cm^2. (T_p of 150 °C and P_p of 129 kPa) **Supplementary Figure 22(b)** presents high-magnification cross-sectional TEM images from different regions of the Ga_2O_3 nanosheet from different regions, indicated by rectangles (1), (2), and (3) in **Supplementary Figure 22(a)**. **Supplementary Figure 22(c)** shows the SAED patterns corresponding to regions (1), (2), and (3) in **Supplementary Figure 22(a)**, indexed to the planes of β-Ga_2O_3. These results confirm that the Ga_2O_3 nanosheet, prepared by pressure-assisted liquid-metal printing, exhibits a crystalline β-Ga_2O_3 phase consistently across various locations over a large area (on the order of a few μm).

For comparison, **Supplementary Figure 23(a)** shows a low-magnification cross-sectional TEM image of a Ga_2O_3 nanosheet synthesized *via* the LMP route at 150°C without applied pressure (Tp = 150°C, Pp = 0 kPa), with the sample fabricated using FIB tool. **Supplementary Figure 23(b)** presents high-magnification cross-sectional TEM images of the Ga_2O_3 nanosheet from different regions, indicated by rectangles (1), (2), and (3) in **Supplementary Figure 23(a)**, revealing no lattice-ordered structure in the LMP-grown nanosheet. **Supplementary Figure 23(c)** shows the corresponding SAED patterns from rectangles (1), (2), (3), and (4) in **Supplementary Figure 23(a)**, confirming the presence of a halo pattern. These results indicate that the GaO_x

nanosheet, prepared by liquid-metal printing without applied pressure, remains in an amorphous phase consistently across various locations over a large area (on the order of a few um).

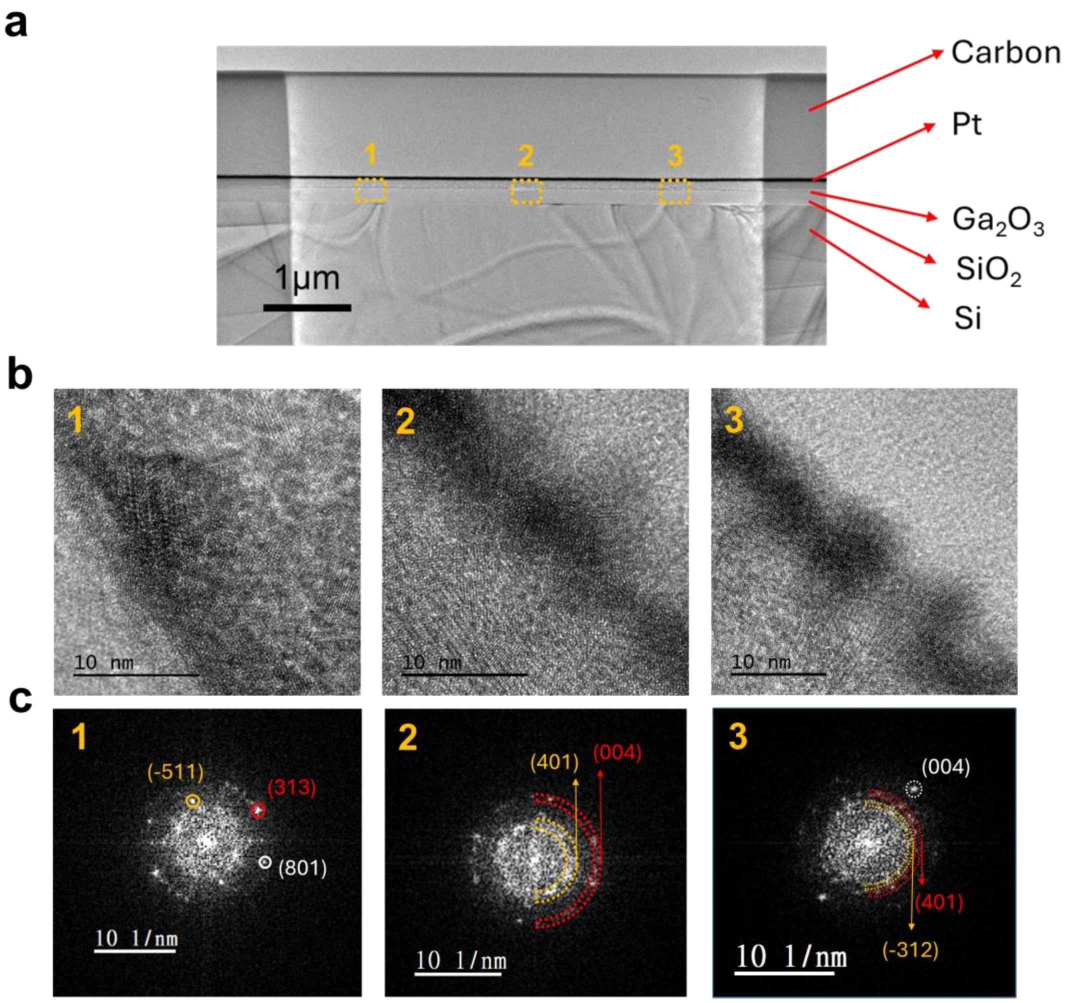

Supplementary Figure 22. (a) Low-magnification cross-sectional TEM image of a sample fabricated using FIB for the Ga_2O_3 nanosheet synthesized by the PA-LMP route with a T_p of 150 °C and a P_p of 129 kPa. (b) Corresponding cross-sectional HRTEM image. (c) Corresponding SAED patterns from regions (1), (2), and (3), respectively.

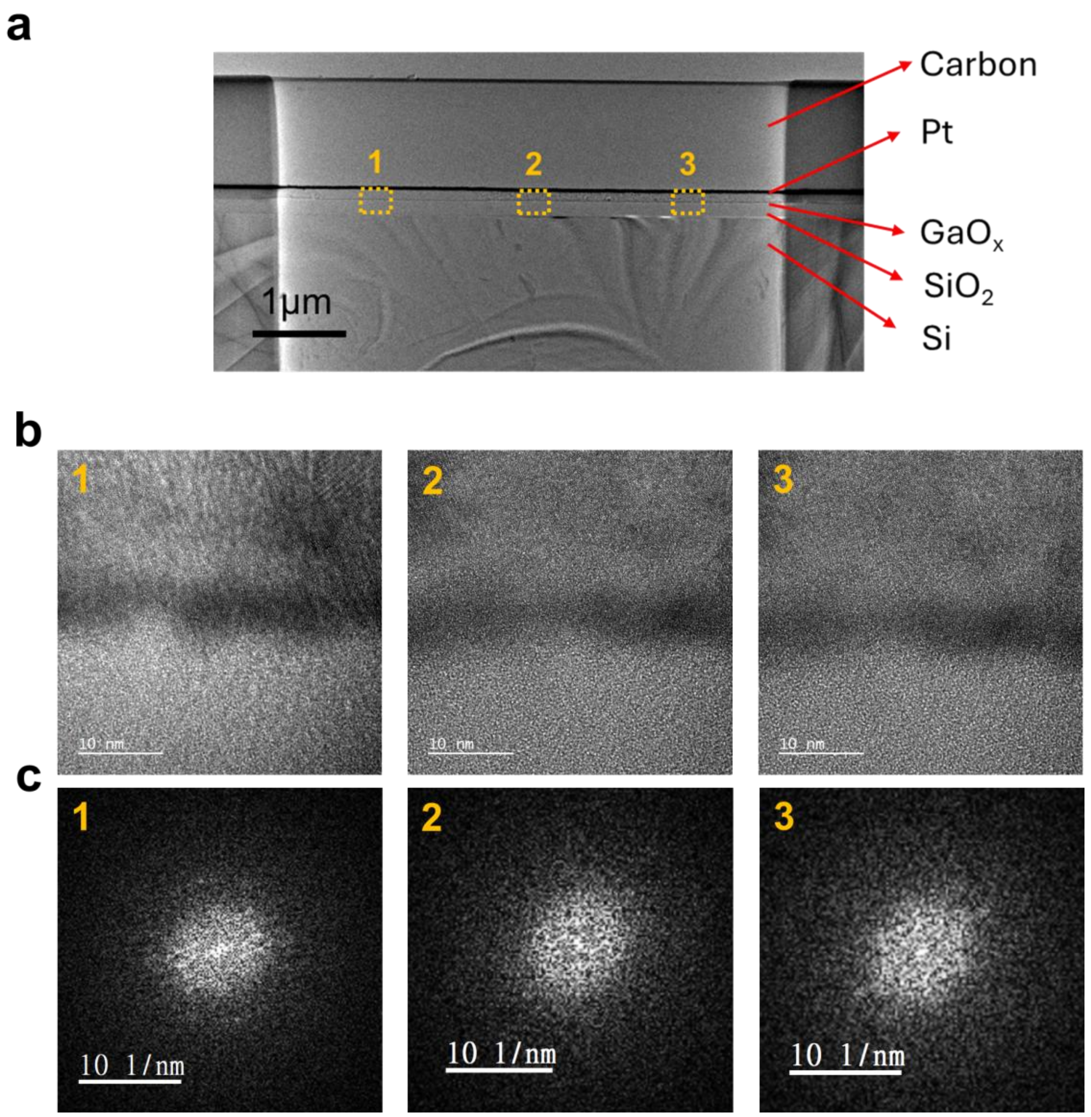

Supplementary Figure 23. (a) Low-magnification cross-sectional TEM image of a sample fabricated using FIB for the GaO_x nanosheet was synthesized using the conventional liquid-metal printing route. (without applied printing process pressure) (b) Corresponding cross-sectional HRTEM image. (c) Corresponding SAED patterns from regions (1), (2), and (3), respectively.

XPS characterization for the Ga_2O_3 nanosheet grown by pressure-assisted liquid-metal printing

Supplementary Figure 24 presents the X-ray photoemission spectroscopy (XPS) spectra of the Ga 3*d* and O 1*s* core level spectra for the Ga_2O_3 nanosheet grown by pressure-assisted liquid-metal printing. (Ga_2O_3 nanosheet synthesized by the pressure-assisted liquid-metal route with the T_p of 150 °C and the P_p of 129 kPa.) For the XPS sample, we fabricated the Ga_2O_3 nanosheet on Si substrate to avoid the O signal from the SiO_2 or glass substrate. The Ga 3*d* core level exhibited a peak position of 19.7 eV, corresponding to Ga^{3+} oxidation state. The characteristic peaks observed at 531.5 eV are attributed to the O 1*s* spectra of the Ga(III)-O bond in Ga_2O_3.

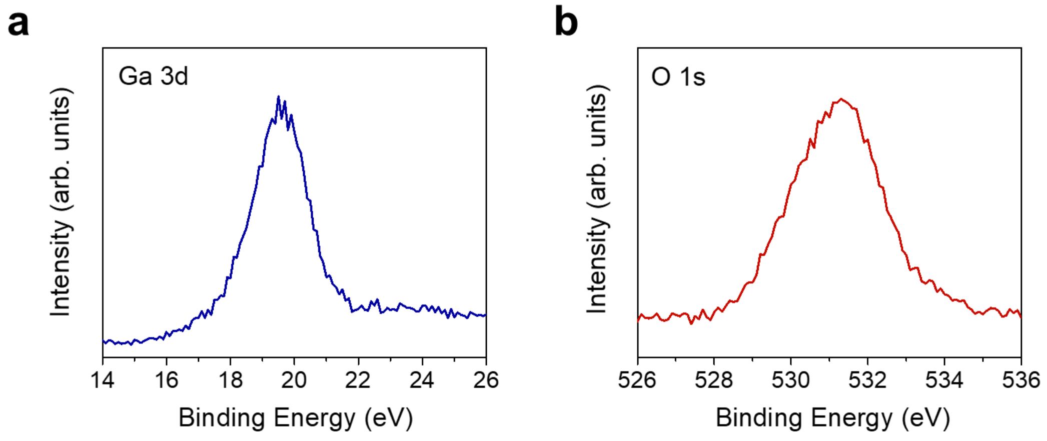

Supplementary Figure 24. XPS spectra of (a) Ga 3*d* and (b) O 1*s* core levels for the Ga_2O_3 nanosheet. (Ga_2O_3 nanosheet synthesized by the pressure-assisted liquid-metal route with the T_p of 150 °C and the P_p of 129 kPa.)

Nucleation energy barrier of the unconfined and the confined nucleation model

In classical nucleation theory, a free energy change per molecule (ΔG) is assumed to be the driving force for nucleation. For the formation of a nucleus, ΔG is given by the sum of the bulk (ΔG_b) and surface (ΔG_s) energy terms, which can be defined as follows[14]

$$\Delta G = \Delta G_b + \Delta G_s = -\left[\frac{\frac{4}{3}\pi r^3}{V_m}\right] k_B T \sigma + 4\pi r^2 \gamma$$

where V_m is the volume per molecule of the nucleus, k_B is the Boltzmann constant, T is the temperature, σ is the supersaturation, γ is the interfacial energy. Low-temperature crystallization can be achieved through highly confined nanogap regions (i.e., nanoscale confinement), which has the potential to lower the energy barrier by reducing the reactive surface area of nuclei, decreasing the surface energy penalty and altering the nucleation pathway by lowering the total energy barrier (**Supplementary Figure 25**).[15, 16] For the two-dimensional crystallization in nanoscale confinement, when the morphology of the nucleus is assumed as the disc shape, ΔG in classical nucleation theory can be rewritten as[15]

$$\Delta G = \Delta G_b + \Delta G_s = -\left[\frac{\pi r^2 h}{V_m}\right] k_B T \sigma + 4\pi r h \gamma$$

where h is the height of nuclei, which is the distance of the confined nanogap. In this study, the h is estimated as < 5 nm and confined by a nanogap of two substrates under external pressure. The confinement reduces the surface energy contribution to the energy barrier, facilitating the crystallization in the nanoscale confined space. **Supplementary Table 2** presents the derivation of the nucleation energy barrier (ΔG) for the unconfined nucleation and the confined nucleation model in the collagen gap region.

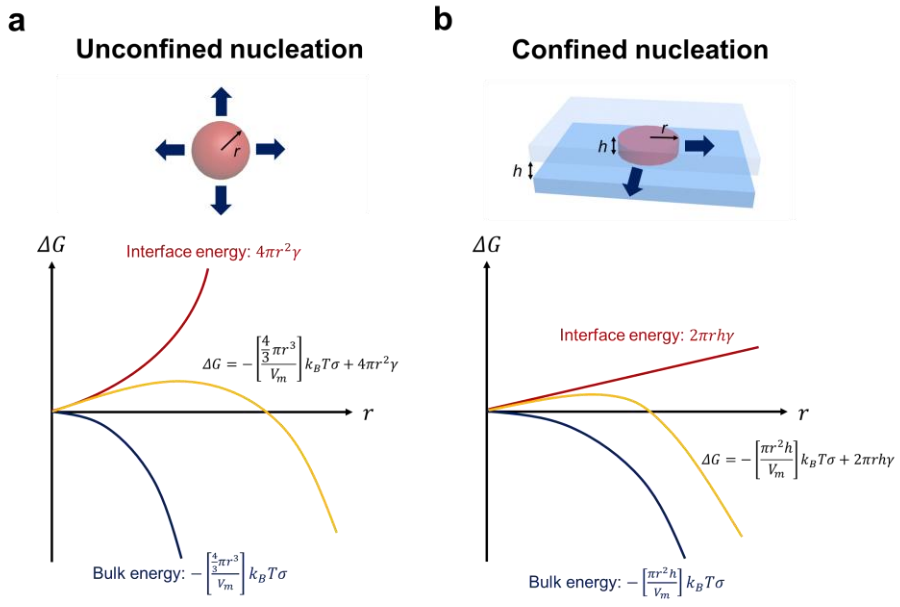

Supplementary Figure 25. Nucleation energy barrier (ΔG) of (a) the unconfined nucleation and (b) the confined nucleation model in the collagen gap region.

Supplementary Table2. Derivation of the nucleation energy barrier (ΔG) of the unconfined nucleation and the confined nucleation model in the collagen gap region.

Parameter	Unconfined nucleation	Confined nucleation
Morphology of nucleus	Sphere	Disc (constant h)
Effective surface area	$4\pi r^2$	$2\pi rh$
Volume	$\frac{4}{3}\pi r^3$	$\pi r^2 h$
$\Delta G = \Delta G_b + \Delta G_s$	$-\left[\frac{\frac{4}{3}\pi r^3}{V_m}\right] k_B T\sigma + 4\pi r^2\gamma$	$-\left[\frac{\pi r^2 h}{V_m}\right] k_B T\sigma + 2\pi rh\gamma$

where V_m is the volume per molecule of the nucleus, k_B is the Boltzmann constant, T is the temperature, σ is the supersaturation, γ is the interfacial energy, h is the height of nuclei, which is the distance of confined nanogap.

Transfer characteristics for linear and saturation region of the Ga_2O_3 nanosheet TFTs

Supplementary Figure 26 shows the transfer characteristics for linear (V_{DS}= 1V) and saturation region (V_{DS}= 20V) of the Ga_2O_3 nanosheet TFTs with different conditions for **Figure 3** in the main manuscript.

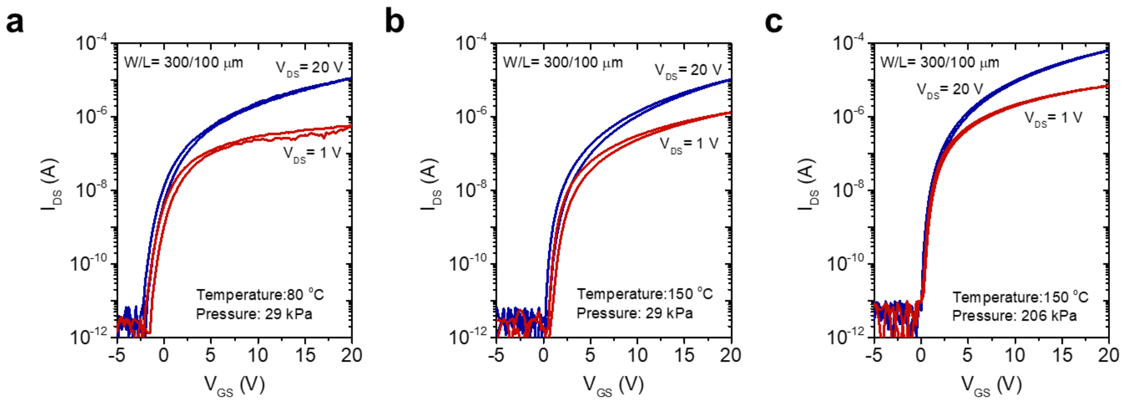

Supplementary Figure 26. Transfer characteristics for linear (V_{DS}= 1V) and saturation region (V_{DS}= 20V) of the Ga_2O_3 nanosheet TFTs with different conditions: T_p of 80 °C and P_p of 29 kPa. (b) T_p of 150 °C and P_p of 29 kPa. (c) T_p of 150 °C and P_p of 129 kPa.

TEM characterization for Ga-rich GaO_x nanosheet grown by pressure-assisted liquid-metal printing

Our observations suggest that the low T_p of 30 °C under the P_p of 29 kPa may not be sufficient to form Ga_2O_3 phases using the PA-LMP method, resulting in a high-density Ga metal impurity and highly conductive channels in the as-prepared device.

Here, we performed transmission electron microscopy (TEM) to investigate the material characterization of the GaO_x nanosheets synthesized by the pressure-assisted liquid-metal route with T_p of 30 °C and P_p of 29 kPa. (**Supplementary Figure 27**) From the SAED, we confirmed that the GaO_x is in the amorphous state. (**Supplementary Figure 27(b)**) However, we found residual metallic Ga embedded in the GaO_x nanosheets. (**Supplementary Figure 27(c)**) The HRTEM image also showed the crystal lattice structure with internal spacings of ~0.276 nm, which are assigned to the (333) plane of the crystal structure of *γ*-Ga metal. (**Supplementary Figure 27(d)**)

In addition, corresponding EDX chemical composition mapping was performed to investigate the elemental distribution. (**Supplementary Figure 28**) The EDX analysis showed an atomic ratio of the GaO_x nanosheet of Ga:O = 58.2% : 41.8%, indicating that the GaO_x is Ga-rich. (**Supplementary Figure 27(d)**)

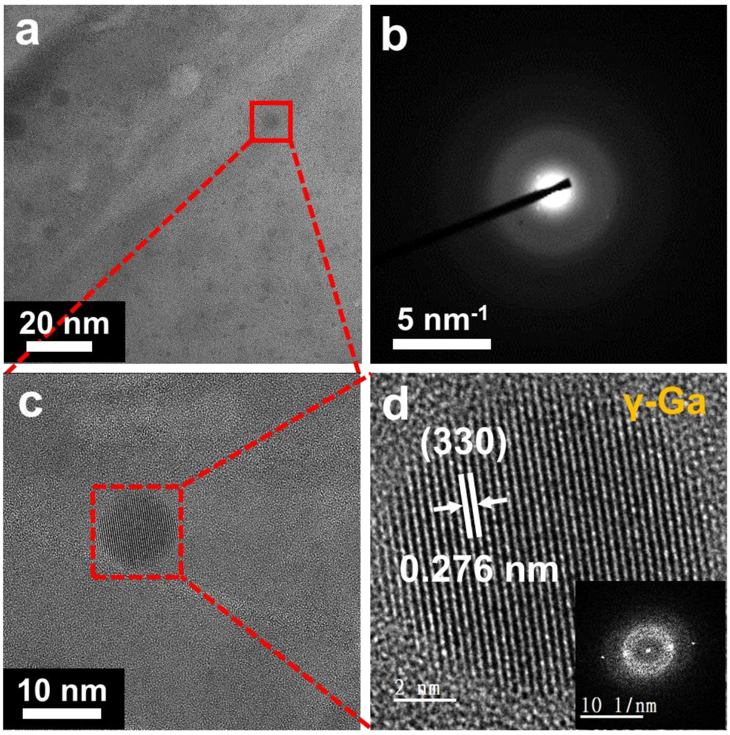

Supplementary Figure 27. TEM characterization of GaO_x nanosheet synthesized by the PA-LMP route with T_p of 30 °C and P_p of 29 kPa. (a) Low-magnified HRTEM images of GaO_x nanosheet. (b) Corresponding SAED pattern. (c) HRTEM image for the Ga metal region (d) Corresponding high-magnified HRTEM image and diffraction pattern extracted by the fast Fourier transform. (inset)

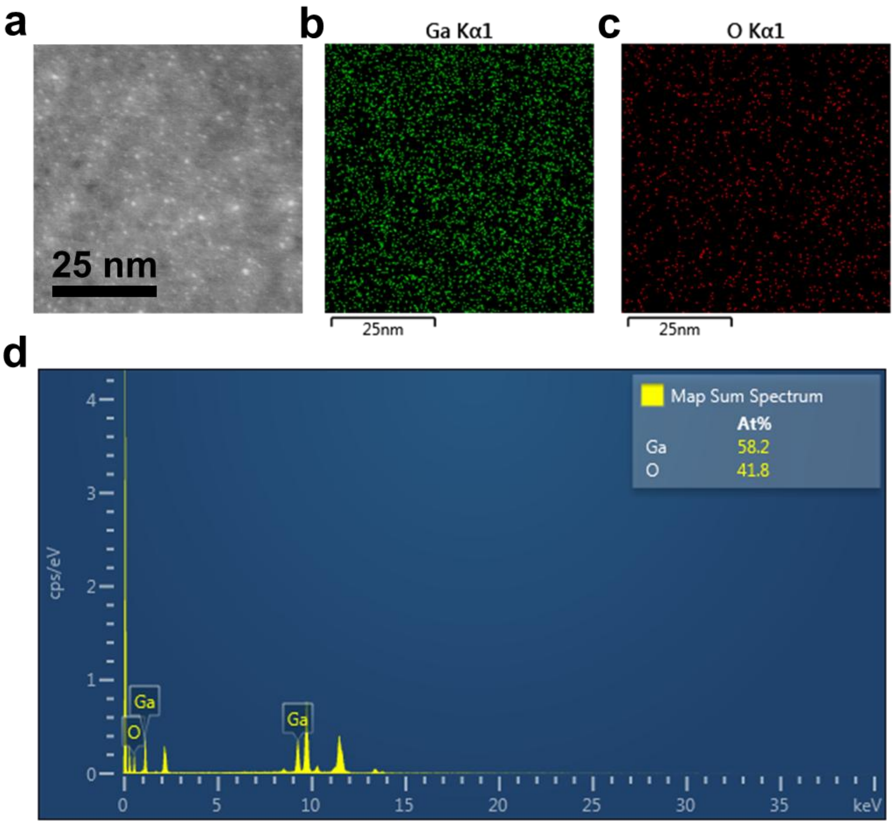

Supplementary Figure 28. (a) Scanning transmission electron microscopy (STEM) image of GaO_x nanosheet (T_p of 30 ^oC and P_p of 29 kPa) and the corresponding EDS composition mappings of (b) Ga and (c) O. (d) Corresponding EDX spectrum for the GaO_x nanosheet. The inset shows the atomic ratio of the Ga_2O_3 nanosheet of Ga:O = 58.2% : 41.8 %.

p-type transport behavior for the GaO_x nanosheet TFT after post-thermal annealing under different atmosphere

Supplementary Figure 29 shows the transfer characteristics for the pristine GaO_x TFTs grown using PA-LMP at a T_p 30 °C and P_p of 29 kPa and the device annealed under different atmospheres, including air, vacuum, and H_2 (forming gas). When the GaO_x channel was grown using PA-LMP at a T_p 30 °C and P_p of 29 kPa, the devices exhibited weak *p*-type field-effect modulation after post-thermal annealing regardless of the annealing atmosphere.

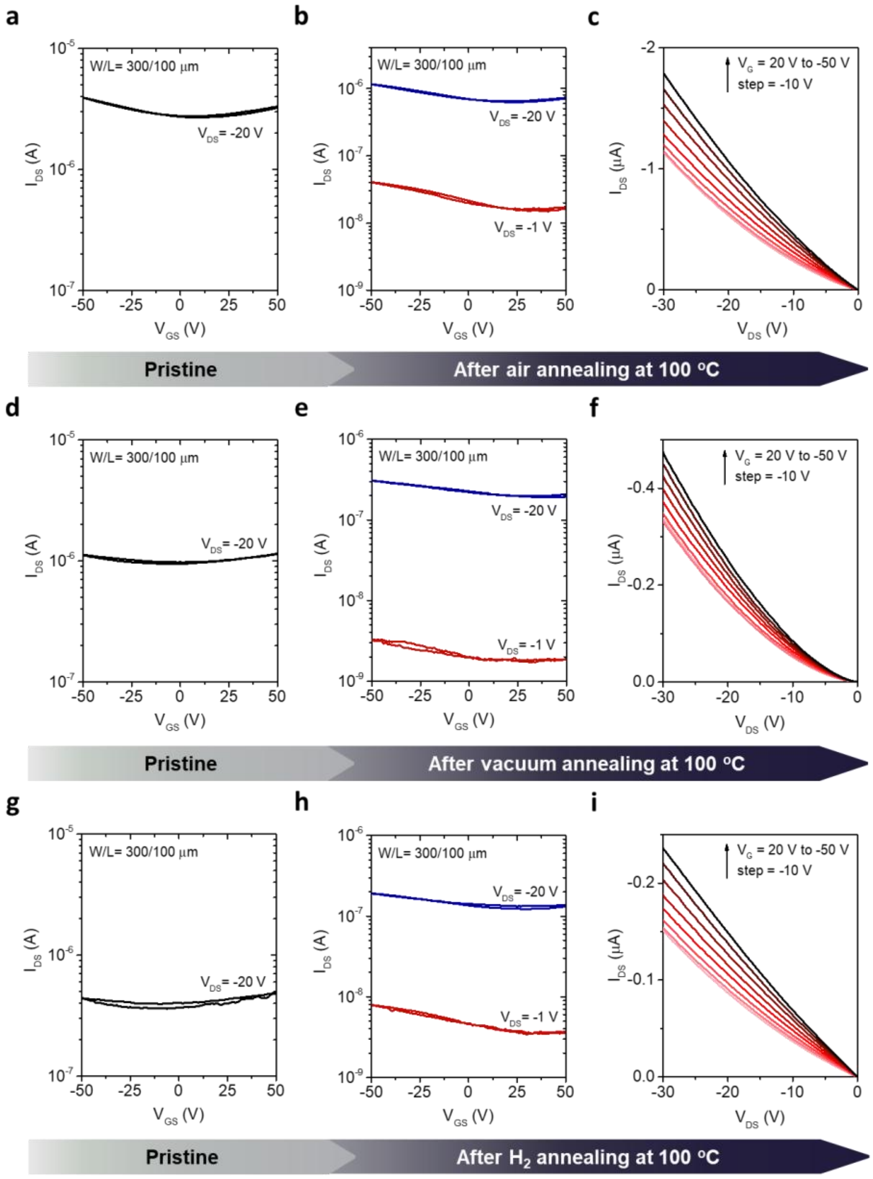

Supplementary Figure 29. *p*-type transport behavior in GaO_x channel grown using PA-LMP at a low process temperature. (GaO_x was grown using PA-LMP at a T_p 30 °C and P_p of 29 kPa) Transfer characteristics for (a) the pristine device and (b) the air-annealed devices at 100 °C. (c) Corresponding output characteristics. Transfer characteristics for (d) the pristine device and (e) the vacuum-annealed devices at 100 °C. (f) Corresponding output characteristics. Transfer characteristics for (g) the pristine device and (h) the hydrogen-annealed devices at 100 °C. (i) Corresponding output characteristics.

Printing process temperature dependence for the Ga_2O_3 nanosheet grown by conventional LMP method

Supplementary Figure 30 shows the transfer characteristics for the Ga_2O_3 nanosheets fabricated by the conventional LMP at different temperatures during the printing process, ranging from 30°C to 200°C. All the devices showed field-effect modulation, indicating that the LMP-grown GaO_x is electrically insulating regardless of the process temperature.

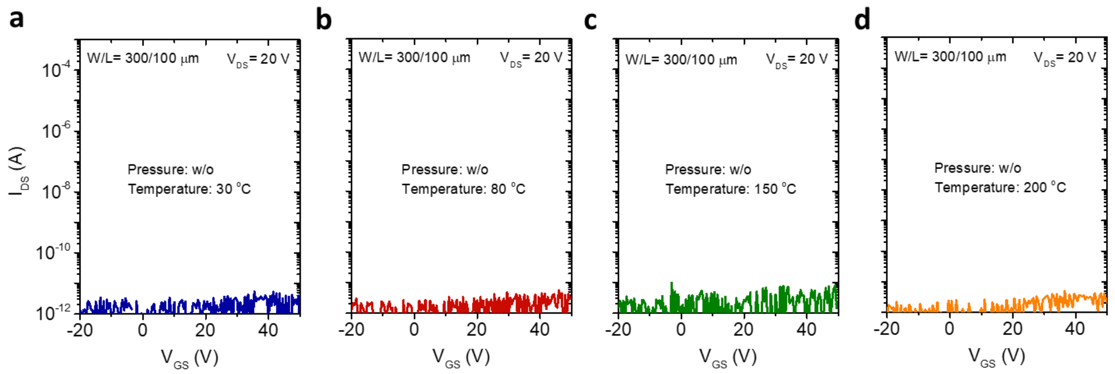

Supplementary Figure 30. Transfer characteristics of the galium oxide nanosheet prepared by the conventional LMP with different process temperatures at (a) 30 °C, (b) 80 °C, (c) 150 °C, and (d) 200 °C.

Hysteresis characteristics in transfer characteristics for the Ga_2O_3 TFT grown by PA-LMP with the uniaxial pressure of 29 kPa

Supplementary Figure 31 shows the variation of the hysteresis characteristics of transfer characteristics for the Ga_2O_3 TFTs. For the devices printed at a P_p of 29 kPa, the width of hysteresis (ΔV_{hys}) at I_{DS} of 10^{-9} A was around 1.25 V and 0.5 V for the TFT printed at 80 °C and 150 °C, respectively. We also found that there is no significant difference between the printing process temperatures of 80 and 150 °C. Meanwhile, the Ga_2O_3 nanosheet TFTs fabricated under the high-pressure condition with 129 kPa and high printing temperature of 150 °C showed small ΔV_{hys} of 0.1 V at the I_{DS} of 10^{-9} A.

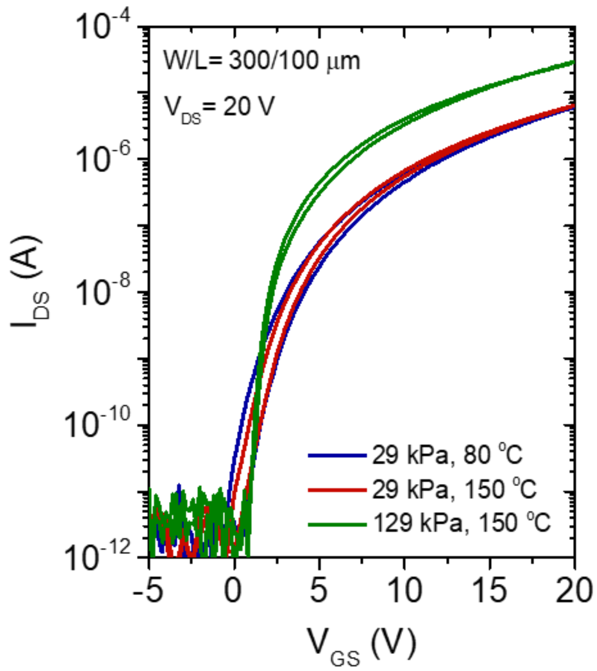

Supplementary Figure 31. Hysteresis characteristics of transfer curves for Ga_2O_3 nanosheet TFTs with different Ga_2O_3 printing conditions.

AFM characterization for the Ga_2O_3 nanosheet grown by pressure-assisted liquid-metal printing

We conducted atomic force microscopy (AFM) analysis on Ga_2O_3 nanosheets on SiO_2/Si substrate samples under different conditions. These samples were prepared based on those investigated for their electrical properties in **Figure 3**.

(a) Process temperatures of 80°C with different printing process pressures (without applied pressure, 29 kPa, and 129 kPa). (See **Supplementary Figure 32**)
(b) Process temperatures of 150°C with different printing process pressures (without applied pressure, 129 kPa, and 206 kPa). (See **Supplementary Figure 33**)

We observed that there was no significant difference in thickness between samples without applied pressure and those subjected to low pressure. However, with increased pressure, we observed a slight decrease in thickness. Regarding roughness, we observed that the surface became flatter for samples subjected to pressure. Additionally, the roughness decreased as the applied pressure increased. Note that the roughness calculation excludes the region with particles. The particles might come from the environment.

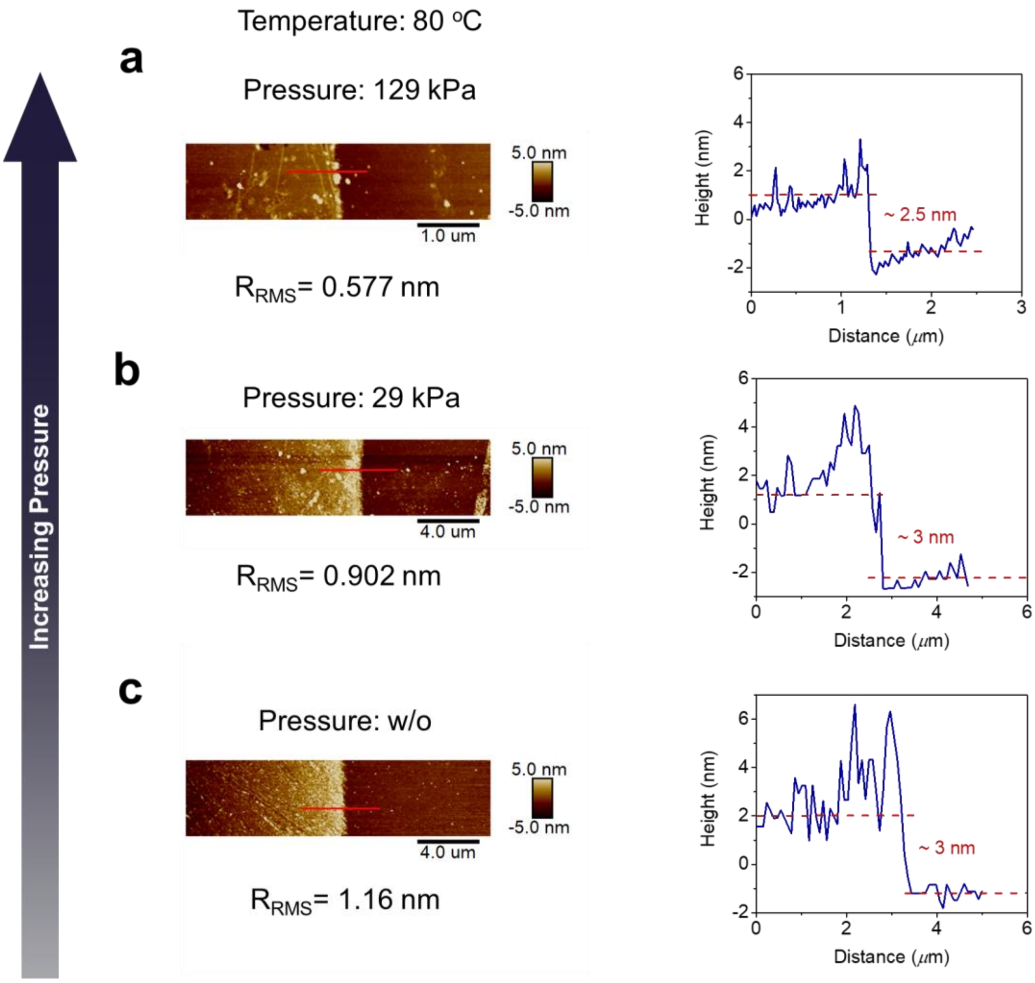

Supplementary Figure 32. AFM image and the cross-sectional step-height profile for the β-Ga_2O_3 nanosheet were prepared by PA-LMP methods at the T_p of 80 °C with different printing process pressure conditions (a) w/o (b) 29 kPa, and (c) 129 kPa.

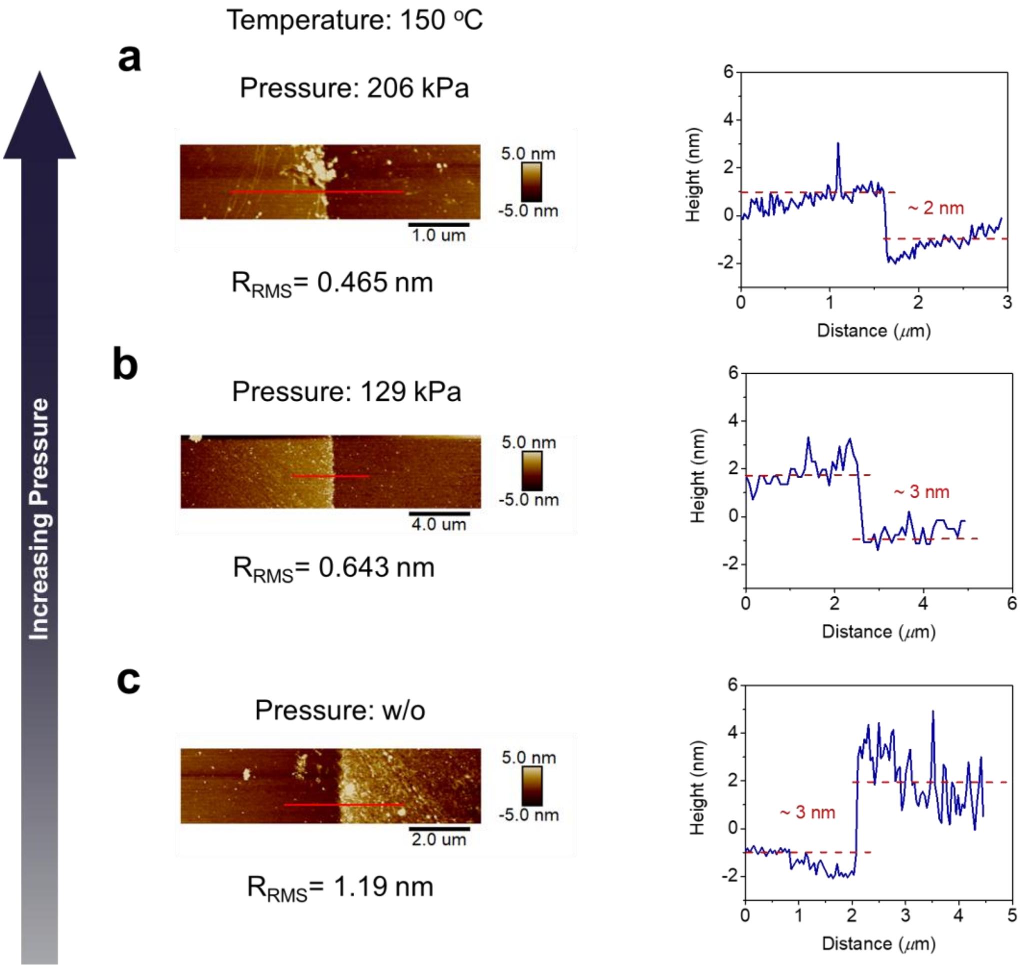

Supplementary Figure 33. AFM image and the cross-sectional step-height profile for the β-Ga_2O_3 nanosheet were prepared by PA-LMP methods at the T_p of 150 °C with different printing process pressure conditions (a) w/o (b) 129 kPa, and (c) 206 kPa.

Summary of TFT characteristics for the PA-LMP grown Ga_2O_3 TFTs

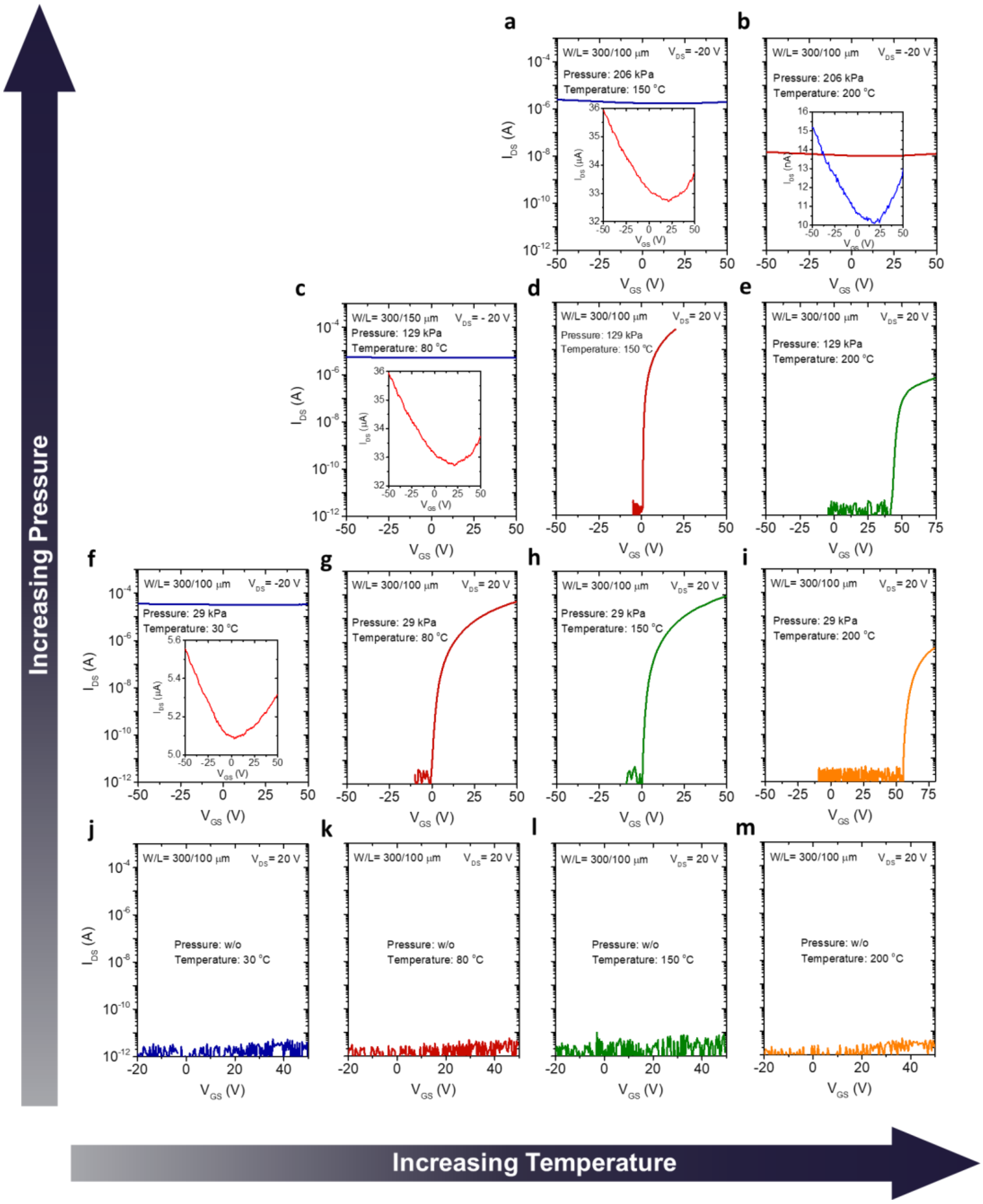

Supplementary Figure 34. Variation of transfer characteristics for the Ga_2O_3 TFTs fabricated by the PA-LMP with different printing temperatures and pressure conditions. The devices are not annealed.

2D TFT simulation parameters for the Ga_2O_3 TFT analysis

Supplementary Table3 TFT simulation parameters for the Ga_2O_3 TFT analysis.

Parameter	80 ºC, 29 kPa	150 ºC, 29 kPa	150 ºC, 129 kPa
Band gap (eV)[17]	4.7	4.7	4.7
Effective DOS for CB (cm^{-3})	3.72×10^{18}	3.72×10^{18}	3.72×10^{18}
Effective DOS for VB (cm^{-3})	1×10^{19}	1×10^{19}	1×10^{19}
Dielectric constant of Ga_2O_3[18]	10	10	10
Electron affinity (eV)	4	4	4
Electron mobility ($cm^2V^{-1}s^{-1}$)	0.9	1.4	12
N_e electron density (cm^{-3})	5.5×10^{14}	5.5×10^{14}	5×10^{14}
N_{GA} (cm^{-3})	2×10^{18}	1.5×10^{18}	5×10^{17}
E_{GA} (eV)	0.15	0.15	0.15
W_{GA} (eV)	0.35	0.3	0.25
N_{GD} (cm^{-3})	0	0	0
E_{GD} (eV)	0	0	0
W_{GD} (eV)	0	0	0

Statistical Analysis of NMOS and CMOS Inverter Performance

We provide the average values of the gain of the inverter circuits. **Supplementary Figure 35(a)** and **(b)** present additional data on the voltage transfer characteristic of the Ga_2O_3 TFT-based NMOS inverter and the *p*-SnO/*n*-Ga_2O_3 TFT-based CMOS inverter. The Ga_2O_3 nanosheets used for the NMOS and CMOS circuits demonstration were grown using a T_p of 150 °C and P_p of 29 kPa . **Supplementary Figure 35(c)** and **(d)** display the average gain of the Ga_2O_3 TFT-based NMOS and *p*-SnO/*n*-Ga_2O_3 TFT-based CMOS inverter at different V_{DD}, respectively. The average voltage gain of the Ga_2O_3 TFT-based NMOS inverters was estimated as 16.9, 45.1, 64.0, 80.5, and 106.4 at V_{DD} from 5 to 25 V, respectively. Meanwhile, the average voltage gain of the *p*-SnO/*n*-Ga_2O_3 TFT-based CMOS inverters was estimated as 27.2, 40.3, 60.7, 90.7, and 124.9 at V_{DD} from 10 to 50 V, respectively.

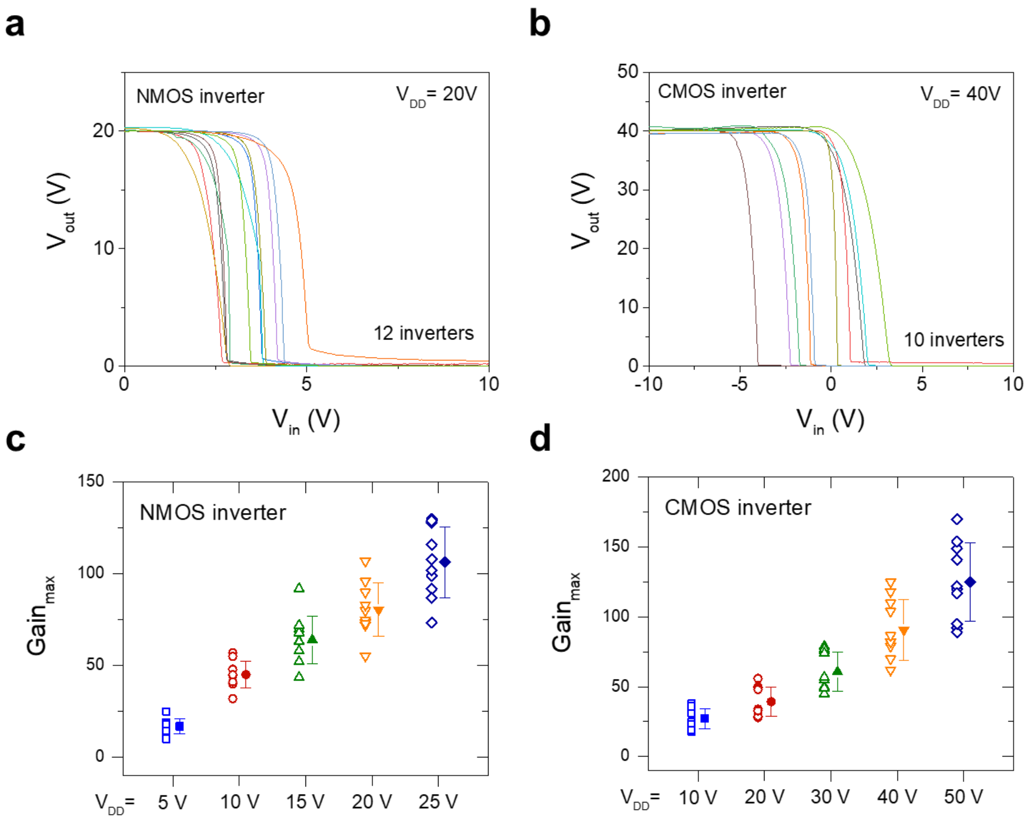

Supplementary Figure 35. (a) Voltage transfer characteristic of the Ga_2O_3 TFT-based NMOS inverter at V_{DD} = 20 V for 12 inverters. (b) Voltage transfer characteristic of the *p*-SnO/*n*-Ga_2O_3 TFT-based CMOS inverter at V_{DD} = 40 V for 10 inverters. (c) Average gain of the Ga_2O_3 TFT-based NMOS at different V_{DD}. The error bars are calculated using data from 12 inverters. (d) Average gain of the *p*-SnO/*n*-Ga_2O_3 TFT-based CMOS inverter at different V_{DD}. The error bars are calculated using data from 10 inverters.

Summary of the device performance for previously reported oxide-TFT-based NMOS inverters

Supplementary Table4. Summary of the reported oxide-TFT-based NMOS inverter performances.

Year	Type of load-TFT	Material	W/L (μm) for load and drive TFTs	Gain	V_{DD} (V)	Noise margin: NM_H / NM_L	Static Power consumption	Ref.
2006	Enhancement	IGO	600/60, 2400/60	1.5	30	--		[19]
2007	Enhancement	a-IGZO	40/10, 822/10	1.7	14	--		[20]
2008	Depletion	ZnO	540/90	4	7	--		[21]
2008		ZnO	500/90	22	2	--		[22]
2009	Depletion	a-IGZO	1000/100, 1000/250	37.4	20	8.2/8.4		[23]
2009	Enhancement	a-IGZO	1000/100, 1000/250	1.6	20	7.5/0.3		[23]
2009	Depletion	ZTO	75, 37.5	10.6	10	2.09/5.9		[24]
2009	Depletion	a-IGZO/ IZO bilayer	50/0.5	15	5	3.5/1.05		[25]
2010	Depletion	ZTO	100/10	-	10	--		[26]
2011	Depletion	a-IGZO	20/20	4.18	10	--		
	Enhancement	a-IGZO	20/20	2.81	10	--		
2012	Depletion	a-IGZO	90/10, 10/10	16.9	10	6/1.7		[27]
2012	Enhancement	a-IGZO	90/10, 10/10	1.9	10	2.5/1.4		
2012	Depletion	a-GIZO/ a-SIZO	250/50	22.5	10	--		[28]
2013	Depletion	ZnO	50/5, 10/5	130	5	--		[29]
2013	Depletion	ZnO	800/50	23	100	--		[30]
2014	Depletion	a-IGZO	20/4	20.5	20	--		[31]
2015	Depletion	a-SZTO/ a-ZTO	250/50, 250/50	25.22	10	1.2/1.2		[32]
2016	Depletion	In_2O_3	1000/80	45	20	--		[33]
2016	Depletion	ZnO	250/25, 100/25	248	25	--		[34]
2016	Depletion	a-IGZO	1/0.5	14	5	3.53/0.6		[35]
2016	Depletion	a-IGZO	50/20	220	2	--		[11]
2016	Depletion	a-IGZO	400/800	45.9	15	--		[36]
2016	Depletion	a-SZTO	50/50	14.2	15	6.1/2.3		[37]
2016	Depletion	a-IGZO	6/50, 50/20	24	1	--		[38]
2016	Depletion	IZO/ IGZO	80/1000	43.9	2	--		[39]
2017	Depletion	ZnO	5/5	48	10	3.13/1.15		[40]
2017	Depletion	ZnO	250/25, 100/25	382	25	--		[41]
2018	Depletion	ZTO	480/20	3.5	10	--		[42]
2018	Depletion	a-IGZO	100/100	40	10	--		[43]
2019	Depletion	a-SIZO	250/50	12.33	5	--		[44]
2019	Depletion	a-IGZO	2000/60	34	1	46%/48%		[45]
2020	Enhancement	ITO	500/300, 2000/100	7.13	10	--		[46]
2021	Depletion	SnO_2	600/30	6.3	3	--		[47]
2021	Depletion	In_2O_3/IGZO	50/30	112	10	--		[48]
2021	Depletion	SnO_2	300/150	198	40	--	0.12 μW	[49]
2022	Depletion	ITO	25/25, 25/10	18	7	47%/43%	0.46 μW	[50]
2022	Depletion	In_2O_3	60/40 ,8/100	209	2	--	--	[51]
2023	Depletion	Ga_2O_3	300/100	130	25	57.2%	28 nW	This work

*IGO: In–Ga–O a-IGZO: amorphous-In–Ga–Zn–O; ZTO: Zn–Sn–O; a-GIZO: amorphous-Ga–In–Zn–O; a-SIZO: amorphous-Si–In–Zn–O; a-SZTO: amorphous-Si–Zn–Sn–O; a-ZTO: amorphous-Zn–Sn–O; IZO: In–Zn–O; ITO: In-Sn-O.

Dynamic switching characteristics of Ga_2O_3 TFTs-based zero-V_{GS}-NMOS inverter

Supplementary Figure 36(a) shows dynamic responses (V_{IN} (in blue) and V_{OUT} (in red)) for Ga_2O_3 TFT-based NMOS inverters made of the enhancement and depletion mode Ga_2O_3 nanosheet TFTs. The square-wave input pulse at a frequency of 1 kHz was applied. Good inversion and response to the input signal were confirmed in the presented circuits. **Supplementary Figure 36(b) and (c)** also show the transient response at rise time and fall time regimes, estimated as 10 μs and 164 μs for the rise and fall times, respectively.

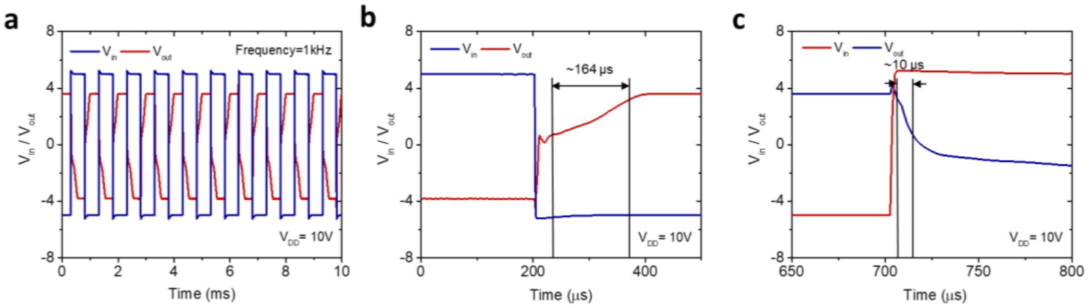

Supplementary Figure 36. (a) Dynamics response measurement at 1 kHz-square-wave input. (V_{DD} = 10 V). The corresponding transient response characteristics for (b) rise and (c) fall times.

Ga2O3 TFTs-based enhancement-type NMOS inverter composed of two enhancement-mode Ga2O3 TFTs

Supplementary Figure 37(a) shows the typical transfer characteristics for enhancement-type NMOS, consisting of two enhancement mode Ga_2O_3 TFTs as the driver and load, respectively. The enhancement-mode TFTs exhibit a μ_{sat} of 2 $cm^2V^{-1}s^{-1}$, *s*-value of 0.28 $V{\cdot}dec.^{-1}$, V_{th} of 6 V, and an on/off current ratio of ~10^7. **Supplementary Figure 37(b)** shows a typical voltage transfer characteristic (VTC) of the NMOS inverter, which represents the V_{out} as a function of the V_{in} with V_{DD}. The inset depicts the equivalent circuit diagram of the enhancement-type NMOS inverter. The output voltage switches from high to low, confirming a clear inverting action with the full-voltage swing. The voltage gain of inverters, which is defined as $-dV_{out}/dV_{in}$ was estimated as 8, 30, and 78 at V_{DD} from 10 to 20 V, respectively (**Supplementary Figure37(b)**). This is comparable with the reported oxide-NMOS inverter (**Supplementary Table4**). **Supplementary Figure 37(c)** illustrates the corresponding supply currents (I_{DD}) as functions of V_{in}. The static currents (V_{in} = 0 V or V_{in} = V_{DD}) are lower than 35 nA, and the static power dissipation, which is defined by $P_{static} = V_{DD}$ $(I_{static\_low} + I_{static\_high})/2$, is 525 nW per logic gate. Also, the P_{out} is estimated as < 0.7 µW per logic gate. The noise margin (NM) is estimated using the maximum equal criterion method, and 47.6 % of the ideal value is obtained (V_{DD}/2). (**Supplementary Figure 37(d)**).

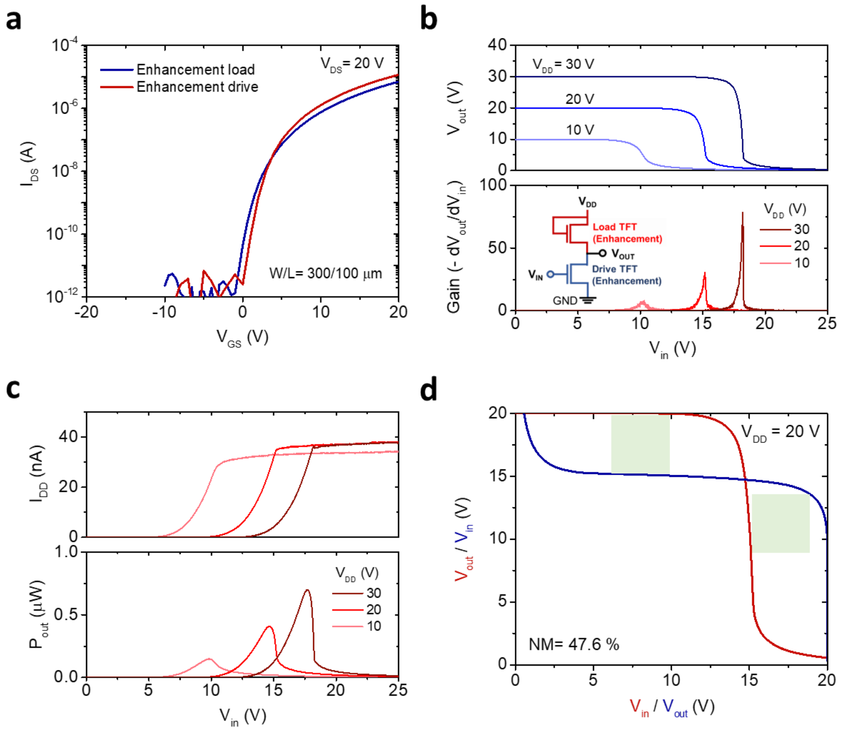

Supplementary Figure 37. (a) Typical transfer characteristics for two enhancement-mode Ga_2O_3 TFTs. for enhancement-type NMOS inverter (b) Top: Voltage transfer characteristic (VTC). Bottom: the corresponding voltage gains of the Ga_2O_3 TFT-based enhancement-type NMOS inverter. Inset is the schematic circuit diagram of the NMOS inverter composed of two enhancement-mode Ga_2O_3 TFTs. (c) Top: the corresponding supply currents (I_{DD}). Bottom: output-power consumption (P_{out}).(d) Noise margin (NM) for the Ga_2O_3 TFT-based enhancement-type NMOS inverter.

Summary of the device performances for the previously reported oxide-TFT-based CMOS inverters

Supplementary Table 5 Summary of the reported oxide-TFT-based CMOS inverter performances.

Year	*p*-material	*n*-material	W/L (μm) for *p*- and *n*-channel TFTs	Gain	V_{DD} (V)	Noise margin: NM_H / NM_L	Static Power consumption	Ref.
2008	SnO_x	In_2O_3	2000/100, 2000/100	11	10	--	--	[52]
2011	Cu_2O	a-IGZO	4000/180, 400/180	120	20	6.01V, 11.68V	--	[53]
2011	SnO	a-IGZO	20.8, 10	4.2	17	9.8 V, 1.0 V	32 pW	[54]
2012	SnO	a-IGZO	-	1.7	15	--	--	[55]
2013	SnO	a-IGZO	1200/650, 256/256	4.9	17	8.6 V, 1.8 V	32 pW	[56]
2014	SnO	a-IGZO	500/100, 500/100	3	10	--	--	[57]
2014	SnO	ZnO	60/30, 30/30	17	10	4.29 V, 4.35 V	--	[58]
2015	Cu_2O	In_2O_3	10/2, 5/10	18	1.5	0.93 V, 0.23 V	1 nW	[59]
2016	SnO	ZnO	200/30, 40/30	12	12	6.2 V/ 3.8 V	--	[60]
2017	SnO	a-IGZO	2000/60, 2000/60	24	40	20 V, 14.4 V	12.5 μW	[61]
2018	SnO	a-IGZO	-	112	10	5.22 V, 3.54 V	--	[62]
2018	SnO	a-IGZO	80/10, 10/10	137	8	3.55 V, 3.43 V	4 μW	[63]
2019	NiO_x	a-IGZO	200/80, 200/80	10.5	12	5.04 V, 5.16 V	--	[64]
2019	Cu_xO	a-IGZO	1000/100, 1000/100	37	60	--	--	[65]
2019	SnO	ZnO	250/100, 100/100	80	10	3.54 V, 3.54 V	0.28 μW	[66]
2019	SnO	a-IGZO	900/10, 150/10	92.4	10	--	--	[67]
2020	Cu_xO	a-IGZO	1000/150, 200/150	14	20	--	--	[68]
2020	SnO	a-IGZO	300/50, 25/50	50	10	--	--	[69]
2020	Cu_2O	ZTO	7200/80, 3600/80	4.2	20	7 V, 6.5 V	--	[70]
2020	Cu_2O	a-IGZO	300/50, 400/150	232	70	--	--	[71]
2021	SnO	a-IGZO	30/20, 30/20	370	10	4.8 V, 4.7 V	--	[72]
2021	SnO	In_2O_3	300/50, 300/50	117	40	--	2nW	[2]
2022	SnO	ITO	25/10, 70/10	62	7	--	16 nW	[50]
2022	Cu_2O	IGZO	500/2, 500/50	62.7	5	0.91V, 0.89V	98.3 nW	[73]
2023	SnO	IGZO	40/20, 40/60	240	70	89.3%	--	[74]
2023	SnO	Ga_2O_3	300/100, 300/100	55	50	--	1 nW	This work

***a-IGZO: amorphous-In–Ga–Zn–O; ZTO: Zn–Sn–O.**

Printing process time effect for the Ga_2O_3 TFTs grown by liquid metal printing

We conducted tests with varying process times under applied pressure for 1, 3, and 5 minutes. (T_P of 150 ^oC and P_P of 29 kPa) However, the mobility remained almost the same, as shown in **Supplementary Figure 38** (selected working device), and no significant change was observed. When we further extended the processing time beyond 10 minutes; however, the Ga_2O_3 channel became insulating, causing the TFT device to cease to operate. Based on these results, we conclude that when the processing time is too long, the carrier concentration of the Ga_2O_3 becomes too low due to a decrease in oxygen vacancies.

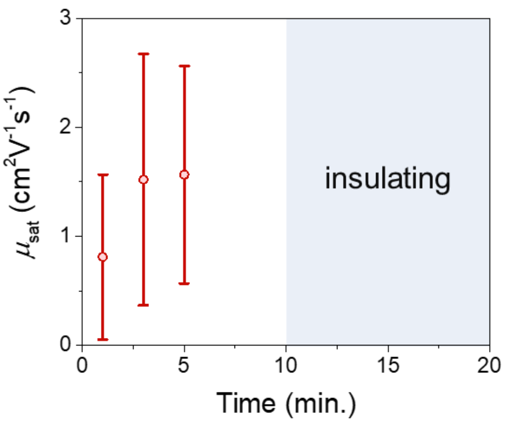

Supplementary Figure 38. Variation of the saturation mobility by different printing process time. A long printing time (>10 min) cannot produce operative Ga_2O_3 nanosheet TFTs. The error bars are calculated using data from 10 representative working devices.)

Supplementary References

1. Zavabeti, A. et al. A liquid metal reaction environment for the room-temperature synthesis of atomically thin metal oxides. *Science* **358**, 332-335 (2017).
2. Huang, C.-H., Tang, Y., Yang, T.-Y., Chueh, Y.-L. & Nomura, K. Atomically Thin Tin Monoxide-Based p-Channel Thin-Film Transistor and a Low-Power Complementary Inverter. *ACS Appl. Mater. Interfaces* **13**, 52783-52792 (2021).
3. Datta, R.S. et al. Flexible two-dimensional indium tin oxide fabricated using a liquid metal printing technique. *Nat. Electron.* **3**, 51-58 (2020).
4. Zavabeti, A. et al. High-mobility p-type semiconducting two-dimensional β-TeO_2. *Nat. Electron.* **4**, 277-283 (2021).
5. Matsuzaki, K. et al. Field-induced current modulation in epitaxial film of deep-ultraviolet transparent oxide semiconductor Ga_2O_3. *Appl. Phys. Lett.* **88**, 092106 (2006).
6. Thomas, S.R. et al. High electron mobility thin-film transistors based on Ga_2O_3 grown by atmospheric ultrasonic spray pyrolysis at low temperatures. *Appl. Phys. Lett.* **105**, 092105 (2014).
7. Kim, J. et al. Conversion of an ultra-wide bandgap amorphous oxide insulator to a semiconductor. *NPG Asia materials* **9**, e359-e359 (2017).
8. Qin, Y. et al. Amorphous gallium oxide-based gate-tunable high-performance thin film phototransistor for solar-blind imaging. *Adv. Electron. Mater.* **5**, 1900389 (2019).
9. Yoon, Y., Kim, M.J., Cho, B.J., Shin, M. & Hwang, W.S. An 8-nm-thick Sn-doped polycrystalline β-Ga_2O_3 MOSFET with a "normally off" operation. *Appl. Phys. Lett.* **119**, 122103 (2021).
10. Purnawati, D., Bermundo, J.P. & Uraoka, Y. Insulator-to-semiconductor conversion of solution-processed ultra-wide bandgap amorphous gallium oxide via hydrogen annealing. *Applied Physics Express* **15**, 024003 (2022).
11. Lee, S. & Nathan, A. Subthreshold Schottky-barrier thin-film transistors with ultralow power and high intrinsic gain. *Science* **354**, 302-304 (2016).
12. Jiang, C. et al. Printed subthreshold organic transistors operating at high gain and ultralow power. *Science* **363**, 719-723 (2019).
13. Wang, G. et al. New Opportunities for High-Performance Source-Gated Transistors Using Unconventional Materials. *Advanced Science* **8**, 2101473 (2021).
14. De Yoreo, J.J. & Vekilov, P.G. Principles of crystal nucleation and growth. *Reviews in mineralogy and geochemistry* **54**, 57-93 (2003).
15. Kim, D., Lee, B., Thomopoulos, S. & Jun, Y.-S. The role of confined collagen geometry in decreasing nucleation energy barriers to intrafibrillar mineralization. *Nat. Commun.* **9**, 1-9 (2018).
16. Meldrum, F.C. & O'Shaughnessy, C. Crystallization in confinement. *Adv. Mater.* **32**, 2001068 (2020).
17. Wang, C. et al. Progress in state-of-the-art technologies of Ga_2O_3 devices. *J. Phys. D: Appl. Phys.* **54**, 243001 (2021).
18. Wang, C. et al. Progress in state-of-the-art technologies of Ga_2O_3 devices. *J. Phys. D: Appl. Phys.* **54**, 243001 (2021).
19. Presley, R. et al. Transparent ring oscillator based on indium gallium oxide thin-film transistors. *Solid-State Electronics* **50**, 500-503 (2006).
20. Ofuji, M. et al. Fast thin-film transistor circuits based on amorphous oxide semiconductor. *IEEE Electron Device Lett.* **28**, 273-275 (2007).

21. Lee, K. et al. ZnO-based low voltage inverter with low-k/high-k double polymer dielectric layer. *Appl. Phys. Lett.* **93**, 193514 (2008).
22. Cha, S.H. et al. ZnO-based low-voltage inverter with quantum-well-structured nanohybrid dielectric. *IEEE Electron Device Letters* **29**, 1145-1147 (2008).
23. Lee, J.-M., Cho, I.-T., Lee, J.-H. & Kwon, H.-I. Full-swing InGaZnO thin film transistor inverter with depletion load. *Japanese Journal of Applied Physics* **48**, 100202 (2009).
24. Heineck, D.P., McFarlane, B.R. & Wager, J.F. Zinc tin oxide thin-film-transistor enhancement/depletion inverter. *IEEE Electron Device Letters* **30**, 514-516 (2009).
25. Yin, H. et al. in 2009 IEEE International Electron Devices Meeting (IEDM) 1-4 (IEEE, 2009).
26. Kim, Y.-H. et al. Ink-jet-printed zinc–tin–oxide thin-film transistors and circuits with rapid thermal annealing process. *IEEE Electron Device Letters* **31**, 836-838 (2010).
27. Cho, I.-T. et al. Full-swing a-IGZO inverter with a depletion load using negative bias instability under light illumination. *IEEE Electron Device Letters* **33**, 1726-1728 (2012).
28. Debnath, P.C. & Lee, S.Y. Full swing logic inverter with amorphous SiInZnO and GaInZnO thin film transistors. *Applied Physics Letters* **101**, 092103 (2012).
29. Li, Y.V., Ramirez, J.I., Sun, K.G. & Jackson, T.N. Low-voltage double-gate ZnO thin-film transistor circuits. *IEEE Electron Device Letters* **34**, 891-893 (2013).
30. Yu, S.H. et al. In/Ga-free, inkjet-printed charge transfer doping for solution-processed ZnO. *ACS applied materials & interfaces* **5**, 9765-9769 (2013).
31. Huang, X. et al. Large-swing a-IGZO inverter with a depletion load induced by laser annealing. *IEEE Electron Device Letters* **35**, 1034-1036 (2014).
32. Han, S. & Lee, S.Y. High performance of full swing logic inverter using all n-types amorphous ZnSnO and SiZnSnO thin film transistors. *Applied Physics Letters* **106**, 212104 (2015).
33. Leppäniemi, J., Eiroma, K., Majumdar, H.S. & Alastalo, A. In_2O_3 thin-film transistors via inkjet printing for depletion-load nMOS inverters. *IEEE Electron Device Letters* **37**, 445-448 (2016).
34. Nayak, P.K., Wang, Z. & Alshareef, H.N. Indium-free fully transparent electronics deposited entirely by atomic layer deposition. *Advanced materials* **28**, 7736-7744 (2016).
35. Lyu, R.-J., Lin, H.-C., Li, P.-W. & Huang, T.-Y. A film-profile-engineered 3-D InGaZnO inverter technology with systematically tunable threshold voltage. *IEEE Transactions on Electron Devices* **63**, 3533-3539 (2016).
36. Wang, M. et al. Threshold voltage tuning in a-IGZO TFTs with ultrathin SnO_x capping layer and application to depletion-load inverter. *IEEE Electron Device Letters* **37**, 422-425 (2016).
37. Han, S. & Lee, S.Y. Full swing depletion-load inverter with amorphous SiZnSnO thin film transistors. *physica status solidi (a)* **214**, 1600469 (2017).
38. Chi, L.-J., Yu, M.-J., Chang, Y.-H. & Hou, T.-H. 1-V full-swing depletion-load a-In–Ga–Zn–O inverters for back-end-of-line compatible 3D integration. *IEEE Electron Device Letters* **37**, 441-444 (2016).
39. Xiao, H. et al. Low-voltage depletion-load inverter using solid-state electrolyte gated oxide transistors. *IEEE Electron Device Letters* **37**, 591-594 (2016).
40. Xu, H. et al. Low-power transparent RFID circuits using enhancement/depletion

logic gates based on deuterium-treated ZnO TFTs. *IEEE Electron Device Letters* **38**, 1383-1386 (2017).
41. Alshammari, F.H., Hota, M.K., Wang, Z., Al-jawhari, H. & Alshareef, H.N. Atomic-Layer-Deposited SnO_2 as Gate Electrode for Indium-Free Transparent Electronics. *Advanced Electronic Materials* **3**, 1700155 (2017).
42. Fernandes, C. et al. A sustainable approach to flexible electronics with zinc-tin oxide thin-film transistors. *Advanced Electronic Materials* **4**, 1800032 (2018).
43. Feng, Z. et al. Fluorination-enabled monolithic integration of enhancement-and depletion-mode indium-gallium-zinc oxide TFTs. *IEEE Electron Device Letters* **39**, 692-695 (2018).
44. Lee, B.H., Kim, S. & Lee, S.Y. Investigation on dependency mechanism of inverter voltage gain on current level of photo stressed depletion mode thin-film transistors. *Solid-State Electronics* **156**, 5-11 (2019).
45. Cai, W., Zhang, J., Wilson, J. & Song, A. Low-Voltage, Full-Swing InGaZnO-Based Inverters Enabled by Solution-Processed Ultra-Thin Al_xO_y. *IEEE Electron Device Letters* **40**, 1285-1288 (2019).
46. Hong, S. et al. Simultaneously Defined Semiconducting Channel Layer Using Electrohydrodynamic Jet Printing of a Passivation Layer for Oxide Thin-Film Transistors. *ACS applied materials & interfaces* **12**, 39705-39712 (2020).
47. Liang, K. et al. Fully-Printed Flexible n-Type Tin Oxide Thin-Film Transistors and Logic Circuits. *Journal of Materials Chemistry C* (2021).
48. Shao, S. et al. Large-area (64× 64 array) inkjet-printed high-performance metal oxide bilayer heterojunction thin film transistors and n-metal-oxide-semiconductor (NMOS) inverters. *Journal of Materials Science & Technology* **81**, 26-35 (2021).
49. Chang, H., Huang, C.-H. & Nomura, K. Low-Temperature Solution-Processed n-Channel SnO_2 Thin-Film Transistors and High-Gain Zero-V_{GS}-Load Inverter. *ACS Appl. Electron. Mater.* **3**, 4943-4949 (2021).
50. Tang, Y., Huang, C.-H. & Nomura, K. Vacuum-Free Liquid-Metal-Printed 2D Indium–Tin Oxide Thin-Film Transistor for Oxide Inverters. *ACS nano* **16**, 3280-3289 (2022).
51. Divya, M., Pradhan, J.R., Priyadarsini, S.S. & Dasgupta, S. High Operation Frequency and Strain Tolerance of Fully Printed Oxide Thin Film Transistors and Circuits on PET Substrates. *Small* **18**, 2202891 (2022).
52. Dhananjay et al. Complementary inverter circuits based on p-SnO_2 and n-In_2O_3 thin film transistors. *Appl. Phys. Lett.* **92**, 232103 (2008).
53. Dindar, A., Kim, J.B., Fuentes-Hernandez, C. & Kippelen, B. Metal-oxide complementary inverters with a vertical geometry fabricated on flexible substrates. *Appl. Phys. Lett.* **99**, 172104 (2011).
54. Martins, R. et al. Complementary Metal Oxide Semiconductor Technology With and On Paper. *Adv. Mater.* **23**, 4491-4496 (2011).
55. Fortunato, E., Barquinha, P. & Martins, R. Oxide Semiconductor Thin-Film Transistors: A Review of Recent Advances. *Adv. Mater.* **24**, 2945-2986 (2012).
56. Martins, R.F.P. et al. Recyclable, Flexible, Low-Power Oxide Electronics. *Adv. Funct. Mater.* **23**, 2153-2161 (2013).
57. Nayak, P.K. et al. Thin Film Complementary Metal Oxide Semiconductor (CMOS) Device Using a Single-Step Deposition of the Channel Layer. *Sci. Rep.* **4**, 4672 (2014).
58. Chiu, I., Li, Y., Tu, M. & Cheng, I. Complementary Oxide–Semiconductor-Based Circuits With n-Channel ZnO and p-Channel SnO Thin-Film Transistors.

IEEE Electron Device Lett. **35**, 1263-1265 (2014).

59. Baby, T.T. et al. A General Route toward Complete Room Temperature Processing of Printed and High Performance Oxide Electronics. *ACS Nano* **9**, 3075-3083 (2015).
60. Li, Y. et al. Flexible Complementary Oxide–Semiconductor-Based Circuits Employing n-Channel ZnO and p-Channel SnO Thin-Film Transistors. *IEEE Electron Device Lett.* **37**, 46-49 (2016).
61. Zhang, J. et al. High Performance Complementary Circuits Based on p-SnO and n-IGZO Thin-Film Transistors. *Materials* **10**, 319 (2017).
62. Li, Y. et al. Complementary Integrated Circuits Based on p-Type SnO and n-Type IGZO Thin-Film Transistors. *IEEE Electron Device Lett.* **39**, 208-211 (2018).
63. Yang, J. et al. Highly Optimized Complementary Inverters Based on p-SnO and n-InGaZnO with High Uniformity. *IEEE Electron Device Lett.* **39**, 516-519 (2018).
64. Chen, C., Yang, Q., Chen, G., Chen, H. & Guo, T. Solution-Processed Oxide Complementary Inverter via Laser Annealing and Inkjet Printing. *IEEE Trans. Electron Devices* **66**, 4888-4893 (2019).
65. Liu, A., Zhu, H. & Noh, Y.-Y. Polyol Reduction: A Low-Temperature Eco-Friendly Solution Process for p-Channel Copper Oxide-Based Transistors and Inverter Circuits. *ACS Appl. Mater. Interfaces* **11**, 33157-33164 (2019).
66. Wang, Z., Kim, H. & Alshareef, H.N. Oxide Thin-Film Electronics using All-MXene Electrical Contacts. *Adv. Mater.* **30**, 1706656 (2018).
67. Joo, H. et al. High-Gain Complementary Inverter Based on Corbino p-Type Tin Monoxide and n-Type Indium-Gallium-Zinc Oxide Thin-Film Transistors. *IEEE Electron Device Lett.* **40**, 1642-1645 (2019).
68. Min, W.K. et al. Switching Enhancement via a Back-Channel Phase-Controlling Layer for p-Type Copper Oxide Thin-Film Transistors. *ACS Appl. Mater. Interfaces* (2020).
69. Lee, A.W., Le, D., Matsuzaki, K. & Nomura, K. Hydrogen-Defect Termination in SnO for p-Channel TFTs. *ACS Appl. Electron. Mater.* **2**, 1162-1168 (2020).
70. Shijeesh, M.R., Mohan, P.A. & Jayaraj, M.K. Complementary Inverter Circuits Based on p-Cu_2O and n-ZTO Thin Film Transistors. *J. Electron. Mater.* **49**, 537-543 (2020).
71. Chang, H., Huang, C.-H., Matsuzaki, K. & Nomura, K. Back-Channel Defect Termination by Sulfur for p-Channel Cu_2O Thin-Film Transistors. *ACS Appl. Mater. Interfaces* **12**, 51581-51588 (2020).
72. Hsu, S.M., Su, D.Y., Tsai, F.Y., Chen, J.Z. & Cheng, I.C. Flexible Complementary Oxide Thin-Film Transistor-Based Inverter With High Gain. *IEEE Trans. Electron Devices* **68**, 1070-1074 (2021).
73. Jung, S.H. et al. Progressive p-channel vertical transistors fabricated using electrodeposited copper oxide designed with grain boundary tunability. *Mater. Horiz.* **9**, 1010-1022 (2022).
74. Kim, H.-M., Choi, S.-H., Lee, H.U., Cho, S.B. & Park, J.-S. The Significance of an In Situ ALD Al2O3 Stacked Structure for p-Type SnO TFT Performance and Monolithic All-ALD-Channel CMOS Inverter Applications. *Adv. Electron. Mater.* **n/a**, 2201202.
